# Live cell imaging of duplex siRNA intracellular trafficking

**DOI:** 10.1093/nar/gkv307

**Published:** 2015-04-13

**Authors:** Markus Hirsch, Mark Helm

**Affiliations:** 1 Institute of Pharmacy and Biochemistry, University of Mainz, D-55128 Mainz, Germany; 2 Institute of Pharmacy und Molecular Biotechnology, University of Heidelberg, D-69120 Heidelberg, Germany

## Abstract

Intracellular distribution of siRNA after *in vitro* transfection typically depends on lipopolyplexes, which must release the siRNA into the cytosol. Here, the fate of siRNAs was monitored by FRET-based live cell imaging. Subsequent to *in situ* observation of uptake and release processes, this approach allowed the observation of a number of hitherto uncharacterized intracellular distribution and degradation processes, commencing with a burst of endosomal releases, followed, in some cases, by fast siRNA influx into the nucleus. The continued observation of intact siRNA against a background of free fluorophores resulting from advanced degradation was possible by a specifically developed imaging algorithm, which identified populations of intact siRNA in pixels based on FRET. This proved to be essential in the end point definition of siRNA distribution, which typically featured partially degraded siRNA pools in perinuclear structures. Our results depict the initial 4 h as a critical time window, characterized by fast initial burst release into the cytosol, which lay the foundations for subsequent intracellular distribution of siRNA. Combination with a subsequent slower, but sustained release from endosomal reservoirs may contribute to the efficiency and duration of RNAi, and explain the success of lipopolyplexes in RNAi experiments in cell culture.

## INTRODUCTION

siRNA-based RNAi has become a powerful tool for studying gene function in vitro and promises to be a versatile tool for *in vivo* application and therapy (1). After the discovery of RNAi, the description of the RNAi pathway and the discovery of siRNA (2–4), the latter became a standard tool for knockdown studies *in vitro* and *vivo*. Typically, synthetic siRNA is delivered by delivery agents of cationic origin typically via endocytosis or related pathways (5–8) and results in the formation of an active RNA induced silencing complex (RISC) that consists of an Argonaute protein as major effector, one strand of the former siRNA duplex (the guide strand), and helper proteins, like TRBP and Dicer (9–11). The siRNA-RISC is localized mainly in the cytosol (12–15) though under certain conditions, loaded RISC was also found in the nucleus (16–19), suggesting the existence of nuclear RNAi targets, or at minimum an Ago dependent nuclear pathway (20–22).

Despite the success of siRNA, an important unsolved question is how and when uptake of, and release from, delivery agents occurs (23), and in how far these features are important properties of successful transfection agents. The development of efficient transfection agents for applications in cell culture and *in vivo* is a highly active field of research (1,24–25), but an understanding of which properties make a transfection agent successful, is slow to emerge. As a motivation for this study, we argue that the kinetics of siRNA uptake and release in the cell are ill understood, and that their investigation using widespread transfection agents is likely to shed light on parameters that contribute to the success of these agents. Beyond the aspect of fundamental research, this may also lead to more efficient agents, and a reduction of material necessary to achieve efficient gene silencing in therapy.

This endeavor requires a near comprehensive tracking of siRNA, both free and bound in lipoplexes, which in turn necessitates fluorescent labeling. Fluorescently labeled oligoribonucleotides (ORN) are established systems to investigate RNA *in vitro*, e.g. in electrophoretic mobility shift assays (EMSA) (26) or single molecule observations (17). Furthermore, single labeled siRNA is commonly used to confirm cellular delivery by verifying the presence in end-point studies in fixed cells (16,27). However, single label studies cannot discriminate between signals originating from the intact, labeled ORN or the free dye, which may occur as a consequence of RNA degradation. One possibility to address this issue is fluorescence correlation spectroscopy (FCS) (17,28–29). This technique, however, is limited to single cell analysis and requires special equipment. Therefore, we established a system for siRNA surveillance based on fluorescence resonance energy transfer (FRET). FRET may be observed between two dyes attached to one ORN (30–32) or to both strands of one siRNA (31,33–37). FRET offers the possibility to survey the integrity of entire populations of siRNA inside cells. Upon RNA degradation and concomitant dissociation of the FRET dye pair, the energy transfer collapses, resulting in lower FRET intensities. Therefore, double labeling of siRNA allows measuring the integrity state of an unknown population of siRNA in both, the cuvette and inside cells. The crucial and informative parameter here is the ratio of FRET to donor emission upon donor excitation; this parameter is called R/G ratio (ratio of red to green fluorescence) (31,34–35,37).

**Figure 1.**
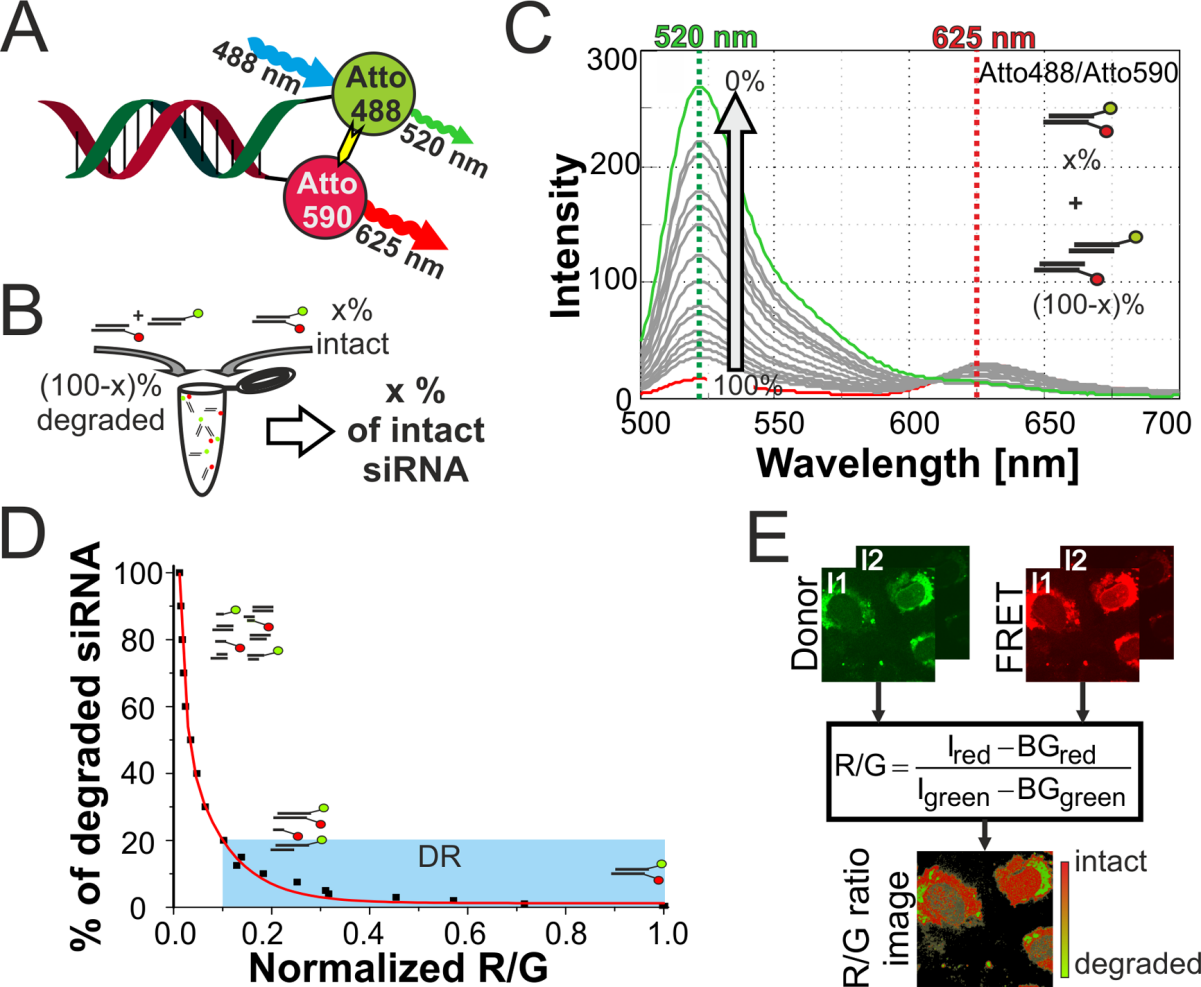
Double labeled FRET siRNA system for integrity measurements. (**A**) siRNA duplex labeled with Atto 488 and Atto 590. (**B**) Different degradation states of siRNA simulated by mixing different ratios of double labeled and single labeled siRNA. (**C**) Emission spectra of siRNA samples with different integrity levels upon 488 nm excitation in the cuvette. Arrow indicates change in donor emission upon decreasing the simulated integrity from 100% intact (red spectrum) to 0% intact (green spectrum). Lines at 520 nm (green) and 625 nm (red) indicate the used intensities for R/G ratio calculations. (**D**) Second-order exponential decay model that describes the relation of R/G ratio with degradation level of siRNA (degradation level = fraction of degraded siRNA). Blue box indicates dynamic range for integrity calculation. (**E**) Schematics of R/G ratio algorithm for determination of integrity level on pixel level basis in confocal imaging data acquired at two different PMT intensities I1 and I2. R/G ratio is calculated as ratio of background (BG) corrected intensities of red (FRET) and green (Donor) emission.

We have previously validated several such FRET-siRNA systems qualitatively (37) and quantitatively (38) using EGFP as a reporter gene. The knockdown was routinely measured 24 h after transfection, and reached close to perfect (>95%) knockdown levels. Although a dye at the 5′ of the antisense strand increases the IC_50_ somewhat, the resulting siRNAs still show IC_50_ values of 700 pM, which is excellent activity and clearly validating the FRET-labeled siRNA as biologically active in the entire RNAi pathway. Knockdown was confirmed in different cell lines and with different dye labels (38,39) after 24 h, delineating a time window in which decisive intracellular delivery and distribution events must take place. Several other groups have employed a number of different dye pairs in the cuvette, in fixed cells and in live cell imaging (32,34,40–42). Early work relied on the classical fluorescein/tetramethylrhodamine (FL/TMR) FRET pair, while more recently developed dyes and dye combinations offer improved spectral properties. In this respect, our recent screening of various FRET pairs identified Atto 488/Atto 590 labeled siRNA duplexes, with Atto 488 as donor dye on the 3′-end of the passenger strand and Atto 590 as acceptor dye on the 5′-end of the guide strand (Figure 1A) as optimized combination with respect to spectral parameters including spectral separation, FRET efficiency, bleed through, cross excitation and photobleaching (35).

Here we use such FRET-labeled siRNA duplexes to study the release of delivered siRNA from lipoplexes formed by siRNA and cationic lipids in living cells. Transfected live cells were imaged by by confocal laser scanning microscopy (CLSM) using FRET settings, and an algorithm was applied to color pixels according to the integrity levels of the siRNA contained. We observed several waves of release events into the cytosol and by determining averaged persistence times of fluorescent labeled intact siRNA in the cytosol, were able to establish a time frame for the availability of large quantities of intact siRNA for RNAi. This suggests that, at least in cell culture, the molecular basis for efficient RNAi relies on intracellular distribution events taking place during the initial 4 h after *in vitro* transfection.

## MATERIALS AND METHODS

### siRNA

Fluorescent labeled siRNA was purchased from IBA GmbH (Göttingen, Germany). siRNA is directed against the mRNA of EGFP (GenBank accession U55762). Passenger or sense strand (s): 5′-GCA AGC UGA CCC UGA AGU UCA U-3′ and guiding or antisense strand (as): 5′-GAA CUU CAG GGU CAG CUU GCC G-3′. siRNA is 3′-labeled with Atto 488 or Fluorescein (FL) dye on the sense strand and 5′-labeled with Atto 590 dye or Tetramethylrhodamine (TMR) on the antisense strand.

### Hybridization procedure for siRNA

siRNA duplexes were formed by hybridization of the respective strands for 1 h at 37°C after an initial denaturation step of 3 min at 90°C. If not other indicated siRNA was hybridized in 1x phosphate buffered saline (1x PBS; 140 mM NaCl, 2.7 mM KCl, 1.5 mM KH_2_PO_4_ and 8.06 mM Na_2_HPO_4_) and 5 μM sense strand. To account for imprecision of siRNA concentration the optimal hybridization ratio of Atto 488/Atto 590 was experimentally determined to be 1:1.2 (s : as) as earlier reported by non-denaturing PAGE analysis (see Supplementary Figure S1A (35)). Fluorescein/Tetramethylrhodamine (FL/TMR) siRNA was hybridized as described in a ratio of 1:1.1 (37). Single labeled duplexes were mixed at a 1:1 ratio. The hybridized duplexes were stored at -20°C.

### Fluorescence emission scans

Fluorescence emission of siRNA samples was measured on a Jasco FP-6500 fluorimeter (Jasco, Tokyo, Japan) equipped with a peltier element for heating and cooling. For routine emission scans, siRNA was diluted to final concentration of 0.1 μM in 1× PBS, and 17 μl were analyzed in 15 μl Suprasil quartz glass cuvettes (Hellma, Müllheim, Germany) with 1.5 mm path length. Emission scans were performed at 25°C with the following parameters: 3 nm bandwidth of excitation and emission, data pitch of 1 nm, response of 0.5 s, scanning speed of 1000 nm/min, and spectral correction of photomultiplier and illumination lamp. Samples were excited at 488 nm, corresponding to donor excitation (Atto 488 and FL) and emission was recorded from 500 nm to 700 nm.

### Integrity simulation & R/G calibration with Atto 488/Atto 590

siRNA samples simulating different integrity states were obtained by mixing double labeled siRNA in different ratios with an equal mixture of donor only and acceptor only labeled siRNA (Figure 1B). A 100% intact sample consists of only double labeled siRNA, while the 0% intact sample consists only of single labeled duplexes. In all samples the same amount of Atto 488 and Atto 590 dye was maintained.

Emission profiles were recorded, background corrected and the R/G ratio was calculated as ratio of donor intensity at 520 nm and FRET emission at 625 nm.

\begin{equation*} {\rm R}/{\rm G} = \frac{{I_{{\rm FRET}} }}{{I_{{\rm donor}} }} = \frac{{I_{{\rm red}} }}{{I_{{\rm green}} }} = \frac{{I_{625} }}{{I_{520} }} \end{equation*}

R/G values of different integrity levels were calculated and normalized to the 100% intact siRNA sample. For calibration of the system the degradation level was calculated.

\begin{equation*} {\rm degradation}\;{\rm level} = 100\% - {\rm integrity}\;{\rm level} \end{equation*}

On the basis of the R/G ratio to degradation level relation a model of second-order exponential decay was fitted (for details see Supplementary Table S1).

### Cell culture

RBE4 (43) cells were cultured under standard cell culture condition in 10% v/v serum (fetal bovine serum), 45% v/v DMEM, 45% v/v HAM's-F10, 100 μg/ml penicillin/streptomycin mix and 1 ng/ml basic fibroblast growth factor (all Invitrogen, Thermo Fisher Scientific, Waltham, USA). Cells were cultured in standard tissue culture treated flasks for maintenance.

### Transfection

One day prior to transfection 20 000 cells were seeded in antibiotic free medium in 8 well μ-slides (Ibidi, Munich, Germany). For transfection 8 pmol (1.6 μl) siRNA was diluted with 29 μl of indicator free DMEM:F12 medium (Invitrogen, Thermo Fisher Scientific) and mixed with 1.7 μl Oligofectamine® diluted with 7 μl DMEM:F12 indicator free medium. After 15 min of incubation 19.5 μl of the transfection mixture was added to the cells, now in 124.5 μl of indicator free medium. In case of a second transfection after 4–6 h, medium was replaced by fresh medium and transfection was performed as described.

### Confocal imaging of Atto 488/Atto 590 siRNA

For live cell transfection μ-slides were placed on a Leica TCS STED CW (Leica, Wetzlar, Germany), equipped with an incubation box, 488 nm, 561 nm and 633 laser lines, 2 HyD detectors and an adaptive focus control system. Image acquisition was started within 1 min after the addition of the transfection mixture. To guarantee live cell conditions, microscope and incubation box were preincubated at 37°C for at least 1 h and cells were incubated in a humidified 5% CO_2_ containing air flow. Images were acquired at 512 × 512 pixel resolution with a 63x oil immersion objective and as z-stacks of five sections at 1μm thickness with a pinhole diameter of 131 μm (1.4 AU). Resulting stacks have a dimension of 246 μm x 246 μm x 5μm X x Y x Z and were acquired in 2 × 2 to 3 × 3 tile scan with an average interval length of 1 to 2 min. For each position, two sequential images were acquired, I1 at a low HyD detector intensity of 10% and I2 at high detector intensity of 100% combined with a transmission image of the 488 nm laser at 25% hardware and 15% software setting. Donor signal between 510 and 540 nm and FRET signal between 605 and 635 nm were recorded simultaneously for a period of up to 8.5 h, consisting of up to 500 scan cycles. For Atto 590 detection, excitation was performed at 561 nm at 5% laser power and the emission was recorded between 605 and 635 nm, similar to the FRET channel.

### R/G ratio algorithm processing of Atto 488/Atto 590

Donor and FRET images of Atto 488/Atto 590 at high (I2) and low (I1) intensity were administered to R/G ratio processing algorithm. Thereby a pixel wise analysis, including thresholding, background correction and color coding of R/G values, of confocal data was performed as earlier published (36,37) with adjustment of the minimal and maximal R/G threshold to 0.5 and 1.2, respectively. The R/G ratio image, with color coded R/G values (R/G > 1.2 in red; R/G < 0.5 in green; 1.2 > R/G > 0.5 in shading from red to green), and the raw data file are used for further analysis.

### Segmentation of cell outlines

On the basis of the transmission and fluorescence data, the cell outline and nucleus were manually segmented with ImageJ ROI manager.

### Integrity distribution

The raw data file (for details see (36,37)) was converted into a 32-bit TIFF image by importing in ImageJ as ‘64-bit real’ image, 512 × 512 pixel, 1 image, no offset, no gap and with option ‘Little-endian byte order’. The intensities correspond to calculated R/G ratios and were extracted to determine the integrity distribution. To discriminate between background and visualized R/G ratios, a cellmask on the basis of the R/G ratio image was created by converting the R/G ratio image into an 8-bit grayscale image and setting all pixel values > 0 to 1. A value of one was added to the raw data before multiplying with the cellmask to discriminate between R/G values of 0 and background pixels. Later, this value was subtracted again to obtain absolute R/G values. Pixel intensities were extracted within predefined ROIs (cell, nucleus or speckles) and grouped into degraded, intermediate and intact fractions (green: R/G <0.5, olive: 0.5<R/G<1.2, red: R/G>1.2).

### Fraction of intact siRNA

The fraction of intact siRNA was obtained as a sum of the products of the pixel fraction and the corresponding integrity level. The integrity level of image R/G values was obtained by dividing the R/G by 5 (normalizing to the average of measured R/G of intact siRNA inside the cuvette) and using the R/G calibration data.

### General event analysis

The time of fluorescence persisting inside the cytosol was determined manually. Release events were sorted according to their time point of release and data plotted in a diagram with sorted event numbers on *y*-axis and time scale on *x*-axis. The length of a horizontal line represents the observed fluorescence time inside the cell. For further analysis only single release events and events showing a depletion within the observation time (full length events) were taken into account.

- Release rates were obtained by fitting a piecewise linear function to the start point of release and event number relation.
- Event duration times were converted into histogram data with a bin size of 5 min and average duration times were obtained by fitting Gaussian populations to local maxima of the distribution.

### Single event analysis

Each cell displaying a release event was segmented into cell and nucleus and mean fluorescence intensity (MFI) values for donor and FRET signal and mean R/G value was measured (ImageJ). An average of all z-slices resulted in the donor, FRET or R/G trace for the observed time point.

## RESULTS

The most common RNAi experiments involving siRNA transfection employ cationic lipids to form lipoplexes which are then taken up by cultured cells by endosomal and related pathways. To visualize the fate of siRNA in such a conventional system, we employed lipoplexes formed from simple mixing of commercial transfection agent and double labeled siRNA. The conditions applied are typical of knockdown experiments in cell culture. The employed concentrations, about 28 nM of siRNA, are a factor of 40 above the IC_50_ value (700 pM) and produced a near complete knockdown in an EGFP-based reportergene assay (38).

### FRET measurements with Atto 488/Atto 590 siRNA

Exploiting the FRET effect between Atto 488 and Atto 590 (Figure 1A), the integrity state can be established from the signals of red and green fluorescence in confocal microscopy. Unlike the FRET efficiency of, e.g. a single molecule, the R/G value does not inform about the distance of the dyes in a molecule, but rather reflects the fraction of siRNA molecules in which the dyes are in close spatial proximity and which are thus considered structurally intact (44). As reported earlier with other dye pairs, we established a calibration curve which relates the R/G ratio to the fraction of intact siRNA (35). R/G values of siRNA in various states of partial degradation were obtained by combining intact double labeled siRNA, with defined amounts of donor-only siRNA and corresponding amounts of acceptor-only siRNA, where the latter two fractions represent the fraction of degraded siRNA (Figure 1B). From the emission spectra obtained upon donor excitation (Figure 1C), the red emission value R was obtained at 625 nm and the green emission value G at 520 nm. A plot of the R/G ratio versus the degradation state yielded a calibration curve (Figure 1D), which we have previously shown to be valid for imaging the integrity state by confocal laser scanning microscopy (CLSM) in cells as well (37). As opposed to the FL/TMR dye pair, the Atto 488/Atto 590 combination displays considerable resistance to photobleaching in the cuvette (35) as well as in cells (Supplementary Figure S2).

For tracing intact siRNA in live cells, an R/G imaging algorithm (36,37) was adapted to the spectral properties of the Atto 488/Atto 590 system. From a calculated R/G ratio for every pixel, the algorithm generates a false color R/G ratio image (Figure 1E) in which volumes are color-coded from red to green according to their calculated content of intact siRNA. This requires data sets generated by two scanning cycles on a CLSM to determine both donor and FRET signal simultaneously at two different PMT intensities (detailed illustration of R/G imaging setup in Supplementary Figure S3A–D). The approach efficiently discriminates siRNA integrity levels from 80% to 100%, but tends to underestimate integrity levels below this threshold, which are colored green in all subsequent figures despite containing up to 80% intact siRNA.

### Live cell observation of *in vitro* transfection with Atto 488/Atto 590 siRNA identifies characteristic release and distribution events

The thus developed system was applied to siRNA imaging in live eukaryotic cells, observing the transfection and release process with cationic lipids as transfection agent in cell culture. Typical experiments started with live cell imaging immediately after addition of the siRNA lipoplex preparation and observed the cells by R/G imaging at 1 min intervals for up to 8 h. During this time span, no detrimental influence of the transfection mixture and irradiation conditions were observed. No increase in dead and/or apoptotic cells was apparent and cells continued to divide after lipoplex uptake (see Supplementary Movie M1).

**Figure 2.**
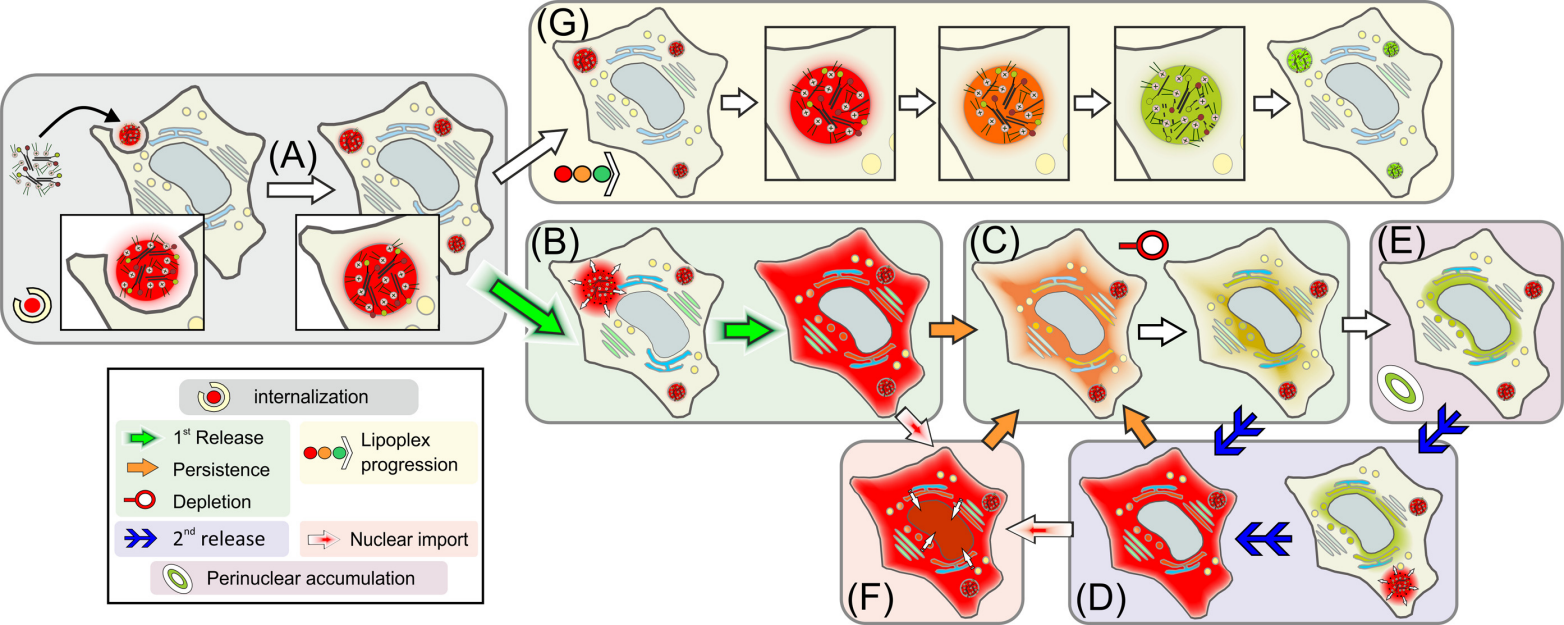
Schematics of observed release event classes. After uptake by internalization (**A**), two alternative fates of labeled lipoplexes were observed: (i) Release into the cytosol (**B**) was followed by depletion from the cytosol (**C**), which was occasionally replenished by repeated release (**D**). After depletion, fluorescent siRNA was observed to accumulate in perinuclear structures (**E**). Occasionally, influx of intact siRNA into the nucleus was observed (**F**). (ii) Lipoplex siRNA lost integrity upon extensive dwelling in endosomal structures without release (**G**). The symbols given in the legend and the designation (A) through (G) will be maintained in all subsequent documentation.

**Figure 3.**
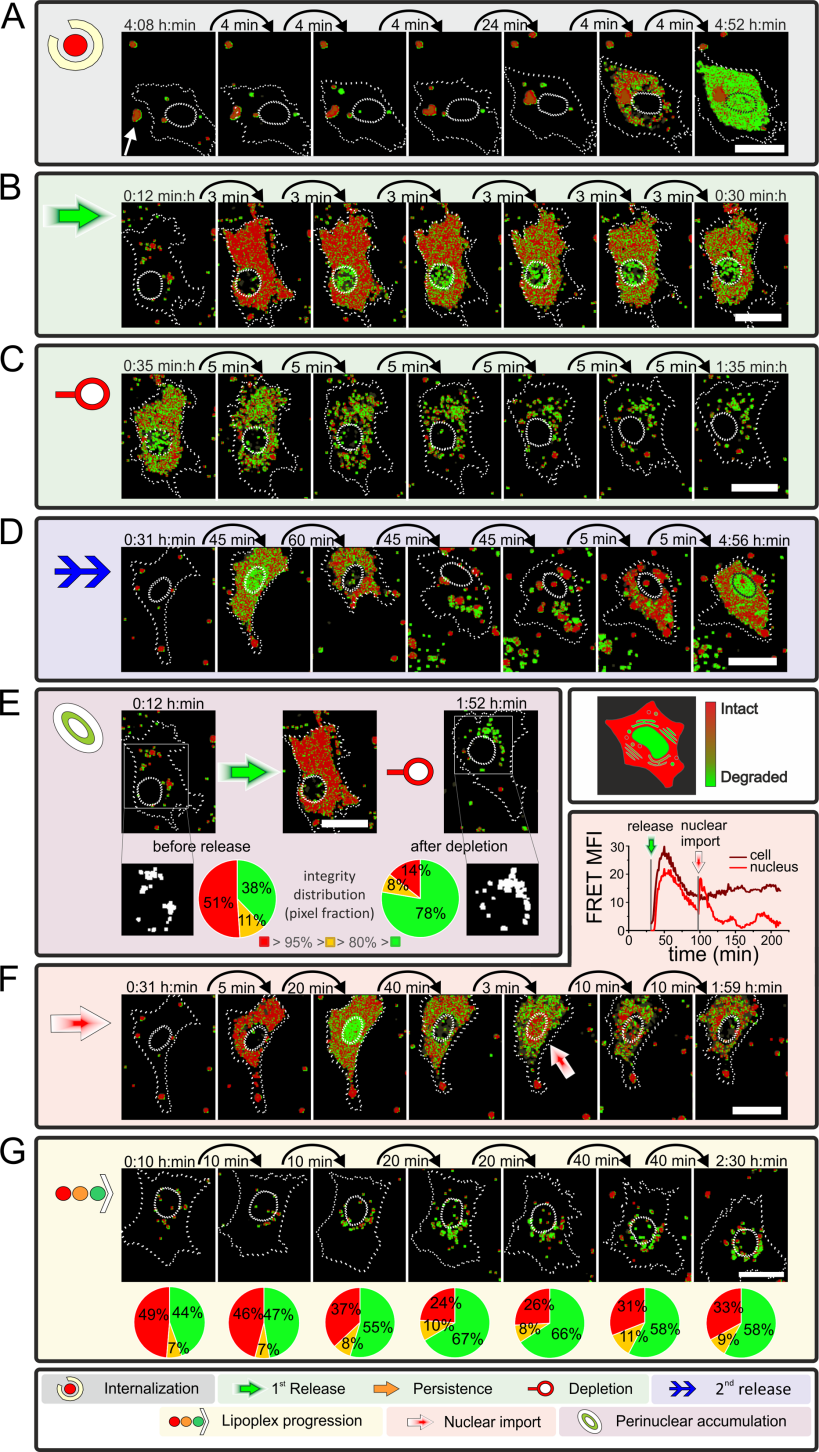
Live cell observation coupled to R/G ratio imaging of exemplary cells illustrating different event classes A–G. Image series depict R/G analyzed data of RBE4 cells transfected with Atto 488/Atto 590. Color of R/G ratio indicates integrity state: red = intact to green = degraded. Time stamp indicates time after transfection. Cell and nucleus are highlighted by dotted lines. Scale bar 25 μm. (**A**) Example of a large lipoplex particle (white arrow) entering a cell that shows a cytosolic release after internalization (see Supplementary Movie M2). A relation between particle and origin of release could not be confirmed nor ruled out. (**B**) Cell showing release of intact siRNA into the cytosol within minutes (see Supplementary Movie M3). Shortly after release, cell displays green pixels inside the nucleus, classified as degraded siRNA. Time series shows a proceeding degradation over time (appearing of olive/green pixels). (**C**) With ongoing observation, a depletion of cytosolic siRNA can be observed (see Supplementary Movie M3). (**D**) Example Cell displaying release & depletion, followed by a secondary release at the end of the series (see Supplementary Movie M4). Secondary released displays intact siRNA present in cytosol. (**E**) Cell before and during release event and after depletion (from left to right). Cellular aggregates (displayed white in ROI-mask) were analyzed according to their pixel composition of intact (red), intermediate (yellow: transition from red to green) and degraded (green) siRNA content (see Supplementary Figure S6 & Movie M3). (**F**) Cell displaying the import of intact siRNA during a release event (red shaded arrow). Time traces in the upper right of the box display the FRET signal during the observed time for the cell and nucleus (see Supplementary Movie M4 & Figure S11). (**G**) Cell showing no cytosolic release but an increase of degraded siRNA in the visible cytosolic aggregates (see Supplementary Movie M5). Integrity distribution is displayed below respective image.

Extensive scrutiny of 5000 min of R/G imaging data obtained during 11 transfection experiments of a total of around 2500 rat brain endothelial cells (RBE4) (43), revealed several classes of distinct pharmacokinetic events. These are schematically depicted in Figure 2 and are each assigned with a key symbol. Following the general presentation of the event classes is a detailed discussion, illustrated by microscopy image figures in which the same key symbols are used (Figure 3A–G). Movie files which contain examples for each class of event have been deposited.

The different classes of events observed are (A) uptake of lipoplex structures containing R/G labeled siRNA, (B) release events characterized by rapid distribution of siRNA in the cytosol, (C) depletion of siRNA fluorescence from the cytosol upon continued incubation, (D) secondary (i.e. repeated) release events in the same cell, (E) accumulation of partially degraded siRNA in perinuclear structures, (F) transport of siRNA into nuclei and (G) persistence of siRNA reservoirs in small, presumably endosomal structures, accompanied by slow decay of the R/G index.

In initial experiments longer imaging periods of up to 3 days were conducted that show releases occur at later time points, too, and that intact siRNA can be found inside cells even after 3 days of incubation (see Supplementary Figure S4).

### Direct observation of siRNA uptake and release (class A and B events)

Fluorescent dots of high R/G ratio corresponding to lipoplex siRNA particles were settled onto the surface of the observations slide, as well as directly onto the cell surfaces, immediately at the beginning of the live cell experiment. In the latter case, internalization events could not be directly observed for lack of a clear discrimination of the inside versus the outside of a cell. However, already at the start of the observation window, fluorescent dots of intact siRNA were also in evidence inside cells, showing internalization to be a fast process. At least some, presumably most of the particles, were internalized by endosomal uptake or related mechanisms, as verified by colocalization experiments of single or double labeled siRNA and LysoTracker® (Supplementary Figure S5).

Direct observation of uptake events (class A event) was possible in those rare cases where moving cells came across a particle on the slide surface. Figure 3A shows such a sequence of images obtained during the uptake of a large lipoplex particle by a moving cell (see Supplementary Movie M2). About 30 min after internalization, siRNA spread throughout the cytoplasm, resulting in a distribution of partially intact siRNA (class B event) within 4 min after what is presumed to be an endosomal rupture. Although the fluorescence was observed to start spreading from that lipoplex particle (see M2), an unambiguous identification of the latter as its origin is made impossible by the presence of additional lipoplexes inside the cell during the uptake process.

While spreading within the cytoplasm, the siRNA R/G index decayed from red toward green, signifying siRNA degradation to a certain degree. Progressive decay of the R/G signal upon continued dwelling in the cytosol was commonly observed in similar events, although the time scale of degradation varied (Figure 3B and Supplementary Movie M3). Release events were observed for more than 13% of all cells. A more detailed numerical analysis is given below, after the presentation of all event classes.

The release as observed in Movie M2 and Figure 3A shows the release of a large amount of labeled siRNA which is observed in some cases. In contrast, the release in Figure 3B or Movie M3 shows the more frequently observed release of a moderate amount of siRNA.

### Depletion from the cytosol and repeated release (class C and D events)

Fluorescence was typically depleted from the cytoplasm within 2 h after a class A event. This depletion (class C event, Figure 3C and Supplementary Movie M3) took 46 min on average, and was significantly slower than the release (*vide infra*). The observation implies that the cytoplasmic concentration of fluorophores dropped below the detection threshold, but it cannot be ruled out that low concentrations of siRNA persist for longer times. Of note, even at the time of depletion from the cytoplasm, most populations displayed an R/G value corresponding to populations of >80% intact siRNA (compare dynamic range of R/G in Figure 1D and Supplementary Figure S3H), showing that our observations still derive from siRNA rather than from dissociated fluorophores. Depletion was observed after 90% of all class A events, with the remainder either due to termination of the experiment, or by a second release event (class D event, Figure 3D and Supplementary Movie M4), causing the cells to show fluorescence over an extended time period. However, the majority of recurrent release events occurred after the cytosol had already undergone a class C depletion. The origin of this second wave of siRNA release may be caused by (i) an immediate release of siRNA from an newly internalized lipoplex, (ii) a release of lipoplexes dwelling in endosomal or lysosomal structures.

### Perinuclear accumulation after release and depletion (class E events)

The disappearance of partially intact siRNA from the cytosol was typically accompanied by a constant increase of fluorescence in small perinuclear structures (class E event). As a typical example, Figure 3E shows a cell containing a population of largely intact siRNA (red pixels) in the cytosol after a class A release. About 1 h later, when the siRNA was depleted (class C) from the cytosol, an increased number of perinuclear structures appeared in fluorescence imaging. While the R/G imaging algorithm depicts 14% of red pixels (integrity above 95%), and 8% of olive pixels (80 to 95% intact), the remaining 78% of pixels are green (Figure 3E). As a reminder, although green indicates significant degradation, up to 80% of the population in a given green pixel may still be intact siRNA. Numerical analysis of pixels with an R/G ratio above 0.5 led to a conservative estimation of still more than 36% intact siRNA in the analyzed region (Supplementary Figure S6). To more closely characterize the intact siRNA content of green pixels, control experiments were designed specifically to confirm, that the R/G ratio interpretation based on cuvette measurements holds true in CLSM experiments even for low R/G values. Mixtures of siRNA of various R/G ratios were transfected and imaged as described. Indeed, as seen in Supplementary Figure S7, the R/G algorithm faithfully detects the fraction of intact siRNA even in mixtures containing as little as 75% intact siRNA. We conclude that the perinuclear structures indeed contained partially intact siRNA, rather than dye molecules dissociated from the RNA. As mentioned initially, practically all cells display fluorescent dots throughout the imaging period, most of which are intact siRNA lipoplexes. However, these dots differ characteristically from the perinuclear dots by their higher siRNA integrity state and more remote location from the nucleus.

In an attempt to further identify these perinuclear structures we performed a staining of mitochondria, lysosomes, ER and Golgi apparatus (Supplementary Figures S8 and S9). While signals clearly do not colocalize with ER and mitochondria, some colocalization is observed with lysosomal compartments, and some more with the Golgi apparatus. The signals colocalizing with lysosomal markers are clearly more remote from the nucleus than the observed perinuclear structures, and presumably reflect early stages of continuously ongoing uptake events. Hence, the colocalization signals with the Golgi apparatus correspond at least in part to the perinuclear accumulation we observed. However, colocalization is still only partial and deeper investigations of this ‘compartment’ are necessary to identify their complete structure.

### Nuclear import of intact siRNA (class F event)

Prompted by the observation of nuclear FRET signals in fixed cells during an earlier phase of our investigations (37) (Supplementary Figure S10), we established a procedure to compare the state of degradation of siRNA populations in different cellular compartments. The workflow, which is detailed in Supplementary Figure S11, essentially involves manual segmentation of the R/G image into nucleus and cytosol, and a calculation of average R/G value for a given compartment from each of the respective pixels. Figure 3F shows an example identified by visual inspection, where a plot of the average FRET signal in the nucleus displays a sharp increase that visibly coincides with a sudden influx of red signal into the nucleus (see Supplementary Movie M4). Like in the cytosol, the fluorescence fades again after the influx. Of note, since the CLSM setup does not have single molecule sensitivity, this observed class F event must originate from simultaneous influx of a large number of siRNA molecules into the nucleus. In addition to the distinctly red signal in the nucleus, we observed numerous occurrences of intense green signals in the nucleus (Figure 3A–D, F). Here again, numeric evaluation of the R/G ratio confirms that intact siRNA rather than single fluorophores are observed (Supplementary Figure S11I).

### Progressive lysis of lipoplexes in endosomes (class G event)

In > 85% of all cells, no release events were observed during up to 6 h of live cell imaging, yet most of the observed cells contained fluorescent lipoplex particles. At least some of these resided in endosomal structures of varying degrees of maturation (Supplementary Figures S5 and S9), which reportedly develop substantial amounts of lytic enzymes, thought to degrade the siRNA payload to a significant degree. Examples of visualization of largely intact siRNA populations from such structures after several hours of live cell imaging implied, that the siRNA was protected against degradation at least for a certain time. Figure 3G shows a time course of siRNA degradation inside a sample structure (see Supplementary Movie M5): the initial state of the ensemble of particle decayed from 49% red pixels to around 33% within 140 min. At that time, the aspect somewhat resembles that of perinuclear aggregates (class E). Overall, the integrity analysis reveals a reservoir of an intact siRNA population within internalized, as yet unreleased lipoplexes, which, despite slow decay, persist at least until the end of the observation phase.

### Statistical analysis of release and persistence after transfection

**Figure 4.**
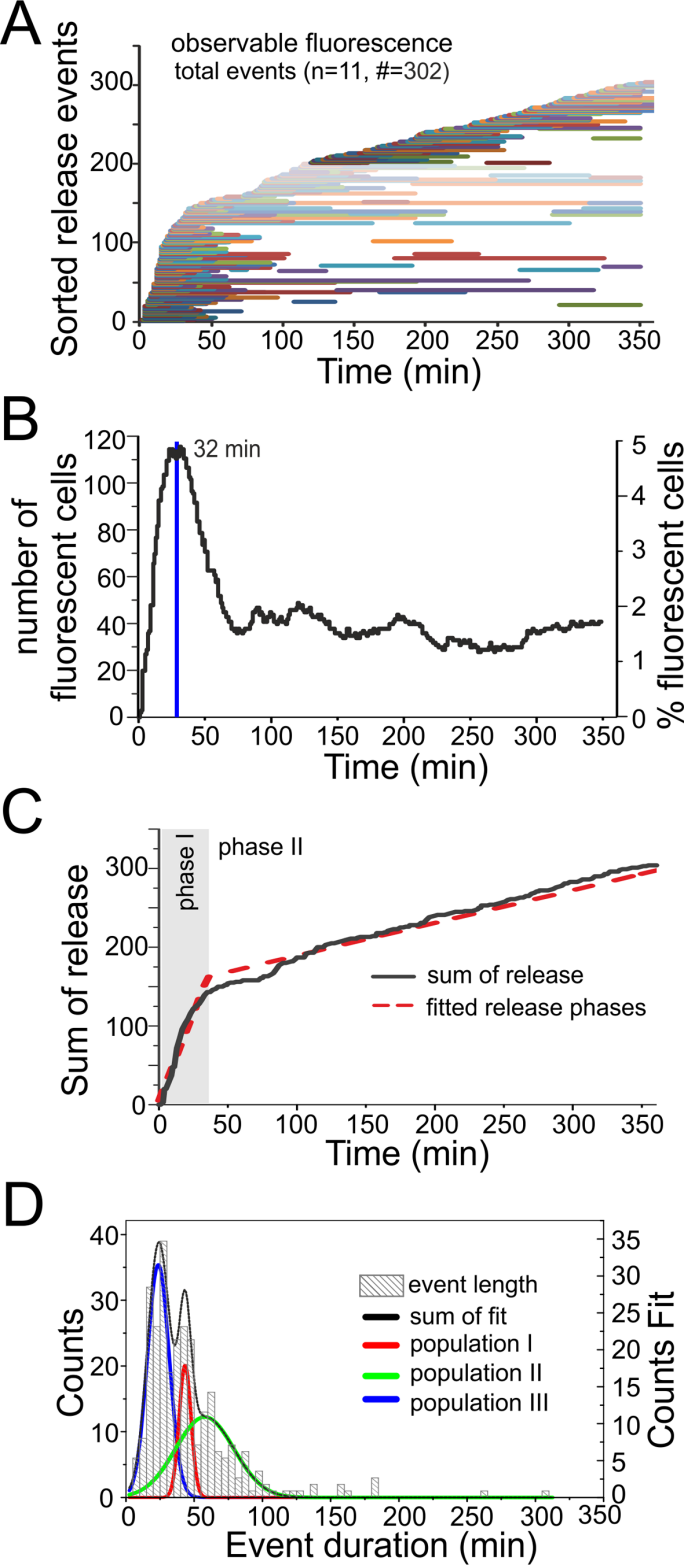
Release analysis of Atto 488/Atto 590 siRNA transfected with cationic lipids. In total 11 live cell transfections were observed resulting in a total of 328 release events, with 302 release events within the first 360 min of observation (see Supplementary Movie M1 for an exemplary experiment). (**A**) Release events in chronological order of onset after transfection. Each event is represented by one horizontal line. Starting point indicates time point of release and the length the persistence time within the cytosol. (**B**) Percentage of fluorescent cells per observed time point after transfection (only full length events were counted). (**C**) Sum of release events over time resulting in two release phases with different release rates. (**D**) Histogram of fluorescence persistence time inside the cytosol before a depletion occurred (only full length events were included). Data were used to determine subpopulations which are indicated in red, green and blue.

A cursory interpretation of class B events suggested that the cells do not display synchronized release behavior at determined time points after transfection, but it was observed that release events clustered during the first hour after addition of the siRNA lipoplexes. For a numerical assessment, all release events occurring during the total of ∼5000 observation minutes were quantified with respect to onset and duration (Figure 4). In total, 54 areas on live cell observation slides (Supplementary Figure S3 for details) containing an average of 44 cells were R/G-imaged and analyzed (see Supplementary Movie M1 as an example experiment). 328 release events (13.8% of all cells) were observed at frequencies between zero and 30 release events (68% of all cells) per area. These numbers indicate strong heterogeneity among the observed cells, and, interestingly, also among the different recorded areas.

Figure 4A summarizes all 302 release events observed within the first 360 min of observation in chronological order of onset time after transfection. Duration of fluorescence in the cytosol is depicted by a horizontal line, whose end marks the time of depletion of siRNA fluorescence from the cytosol. The number of cells with a fluorescent cytosol as consequence of a release event shows a clear maximum number of cell after 32 min, representing ∼5% of all cells (Figure 4B). After ablation of this initial burst, release events and cytosolic fluorescence continued more or less evenly throughout the entire duration of the experiment (Figure 4A), leveling out at a steady state of 1.5–2% of all cells showing fluorescence. Accordingly, a sum plot of the release events as shown in Figure 4C, suggests two different release phases, with an initial short phase of around 30 min in which release events are frequent, (1.9‰ cells per minute show a new release), followed by a second, enduring release rate with a constant moderate release of 0.2‰ cells per minute (Figure 4C).

A histogram of fluorescence persistence times in the cytosol (length of bars in Figure 4A), compiled from full length, first release events, shows typical persistence times below 50 min, with persistence times above 100 min being very rare exceptions (Figure 4D). The most pronounced of three maxima identified by fitting local Gaussian distributed populations, is found at 24 min, with the two others occurring at 43 and 58 min. Given the observation of repeated release events inside the same cell, some of the longer persistence times might be composed of several events.

These observations point out a typical feature of single cell analysis, namely the heterogeneity of a cell population otherwise assumed to respond uniformly to a treatment. Of note, from permutations of early and late onset of release, and different persistence times alone, half a dozen different fates for siRNA in a cell arise.

## DISCUSSION

The optimization of RNAi efficiency and duration will be of continued interest for decades to come. While siRNA application is already extremely efficient and comparatively easy to implement, further improvements will open new fields in life science and therapy. While much of the initial progress in siRNA delivery was owed to try and error with RNAi efficiency as readout, further improvement is likely to come from a detailed understanding of the various events encountered by siRNA during internalization and release in a cell. Using live cell imaging, we observed and identified a number of distinct events that determine the intracellular fate of siRNA molecules after transfection. An improved understanding of the flow of a transfected siRNA population to either degradation or RISC incorporation conceivably might open avenues to manipulate those pathways. The present work was able to significantly advance our understanding of siRNA intracellular distribution because of an extended time frame accessible to live cell observation. This extended time frame was made possible by using FRET-labeled siRNA, which permits an assessment of the integrity/degradation status of the siRNA population in a given pixel of a CLSM image. While single labeled nucleic acids, including siRNA, are routinely observed (45), nucleolytic degradation progresses within the observation time increase the likelihood of observing free dye, which would incorrectly be interpreted as intact siRNA. Here, R/G imaging prevents a progressive loss of confidence with time, and actually succeeds in identifying subfractions of intact siRNA hidden inside pixels containing mainly partially degraded, single labeled RNA fragments.

### Insights from long-term observation of intact siRNA

Some of the observed events, such as endosomal uptake and endosomal rupture, have previously been described by other techniques (45–47). In particular endosomal rupture and subsequent release of siRNA into the cytosol are discussed as key events for RNAi, to which the properties of improved transfection agents must be tailored (23,48–49). Although this endeavor proved extremely laborious, we have undertaken to quantify key features such as onset time and frequency of endosomal release events. As a result that is so far unique in its nature, we documented a biphasic release behavior, with an initial intense release of about 30 min duration, followed by a second phase of much lower basal release frequency.

Snapshots after 1, 2 and 3 days did not reveal any changes with respect to the state between 4 and 5 h after transfection. Thus, by being able to observe the degradation state of an extended timespan, we were able to determine that the most dynamic changes to intracellular distribution occur in the initial ∼60 min after transfection. These therefore constitute a crucial time window, during which the cytosol is flooded with large amounts of siRNA. Possibly, the high concentration of siRNA in many cells during this phase is conductive to chasing endogenous small RNAs from the assembled RISCs, which would make the fast burst release in phase 1 (Figure 4) a critical event for fast and efficient onset of a specific RNAi effect in the ensemble of cells. The sustained slow release in phase 2 (Figure 4) might determine the duration of efficient siRNA over several days.

Other long-term events observed by us, such as siRNA degradation in lysosomes, have been inferred from lysosomal degradation of other biopolymers or merely been speculated upon (12,49–53). Here, only R/G imaging made real-time observation of such events possible, providing a first readout in the search for means of, e.g. therapeutic manipulation.

### Limits of interpretation

Importantly, the FRET-based approach used here does not allow to identify the precise mode of degradation, and can thus not discriminate siRNA incorporation into RISC from degradation by cellular nucleases. As a cautionary note, we point out that Argonaute loading is insufficient to explain the observed dynamics of integrity changes and cellular localization of siRNA in the light of the following numbers reported: approximately 300 siRNA molecules are required for efficient gene silencing (54), an estimated amount of 100 000–1 000 000 siRNA are delivered per cell under our conditions (37), an estimate of 100 000 molecules of the various Argonaute proteins are present per cell (55), and finally many competing endogenous substrates of Argonautes, like miRNA, are present in the cell. Consequently, a significant fraction of the observed siRNA must be degraded by nuclease or helicase activity that is not associated to Argonaute. Therefore, not all events observed here may straightforward be interpreted as part of the RNAi process, rather some generic RNA transport and degradation mechanisms are sure to contribute.

### Outlook

Clearly, the most intriguing and novel observations reported here occurred at advanced time points, and they too, are only made possible by R/G imaging. These other events, including in particular siRNA clearance from the cytosol, and accumulation of partially intact siRNA populations in perinuclear bodies, may be related to events described for other classes of RNAs in cell biology (12,56–58), or be entirely new processes. The plethora of discovered events prevents us from clarifying and quantifying all details in this initial report, but there clearly are many points for subsequent studies.

## SUPPLEMENTARY DATA

Supplementary Data are available at NAR Online.

SUPPLEMENTARY DATA
