# Supplementary material for: Live cell imaging of duplex siRNA intracellular trafficking
Source: Nucleic Acids Res. 2015 Apr 13;43(9):4650–60. doi: 10.1093/nar/gkv307 (PMC4482072; doi:10.1093/nar/gkv307)
Supplement: SUPPLEMENTARY DATA [file supp_43_9_4650__index.html]

Live cell imaging of duplex siRNA intracellular trafficking — SUPPLEMENTARY DATA 

# Live cell imaging of duplex siRNA intracellular trafficking

## SUPPLEMENTARY DATA

**Files in this Data Supplement:**

- SUPPLEMENTARY DATA
- SUPPLEMENTARY DATA
- SUPPLEMENTARY DATA
- SUPPLEMENTARY DATA
- SUPPLEMENTARY DATA
- SUPPLEMENTARY DATA
- SUPPLEMENTARY DATA
