# Supplementary material for: Live cell imaging of duplex siRNA intracellular trafficking
Source: Nucleic Acids Res. 2015 Apr 13;43(9):4650–60. doi: 10.1093/nar/gkv307 (PMC4482072; doi:10.1093/nar/gkv307)
Supplement: SUPPLEMENTARY DATA [file supp_gkv307_nar-03459-y-2014-File006.pdf]

# Live cell imaging of duplex siRNA intracellular trafficking

Hirsch Markus and Helm Mark

## SUPPLEMENTARY DATA

### SUPPLEMENTARY MATERIALS AND METHODS

#### PAGE analysis and purification of siRNA

For verification of the ratio hybridization approach, siRNA duplexes were analyzed on 15% non-denaturing PAGE with respective controls for single and double strands. Fluorescence signals were detected by scanning the gel with a Typhoon 9400 (GE Healthcare, Little Chalfont, UK) at 488 nm excitation and emission filters for donor signal (520 BP 40) and FRET (670 BP 30) by using a beamsplitter at 580 nm. Acceptor excitation was performed at 633 nm excitation and a 670 BP 30 emission filter.
For the gel purification fluorophore labeled siRNA duplex bands were excised after fluorescence scan and siRNA was eluted in 0.5 M $NH_4Ac$ at 4°C over night. siRNA was EtOH precipitated by addition of 2 vol of EtOH, 1 h incubation at -20°C and centrifugation for 1 h at 15 000 xg.

#### Transfection for fixed cell samples

50 000 RBE4 (1) cells were seeded one day prior to transfection on coverslips (Ø 18 mm), coated for 2 h at 37°C in 1% collagen solution (Type I from rat tail; Sigma-Aldrich, St. Louis, USA), in antibiotic free culture medium in a 12 well culture plate. For transfection 30 pmol of 5 µM double labeled siRNA, or sample simulating siRNA at a certain integrity level, was added to 100 µL of Optimem (Invitrogen) and mixed with 6 µL of Oligofectamine diluted with 24 µL Optimem. After 15 min of incubation the mixture was added dropwise to cells and cultivated for 4 h. Before addition of the mixture, medium was exchanged to culture medium without antibiotics and without FBS. For fixation medium was removed, cells washed once with 1x PBS followed by a 10 min incubation with 4 % formaldehyde in 1x PBS. After fixation cells were washed three times in 1x PBS and cells mounted with fluorescent mounting medium (Dako, Hamburg, Germany) onto object slides.

#### Confocal bleaching of Atto 488/Atto 590 siRNA

For analyzing the bleaching behavior under confocal excitation conditions, fixed cell samples of donor only, acceptor only and double labeled siRNA were prepared. The exposition was limited to a 64x64 pixel region by simultaneous 8-fold zoom resulting in same scanning speed as for live cell imaging on the LEICA STED CW. Area was excited with same settings as for live cell imaging resulting in 2000 excitation cycles (1000 scans for each high and low PMT setting). Mean fluorescence intensity (MFI) inside the 64x64 pixel region was determined (ImageJ) and plotted against scan numbers. R/G time traces were calculated as average of FRET to donor MFI.

**Confocal imaging of FL/TMR siRNA**

Fixates FL/TMR siRNA samples were analyzed on a Leica DM IBR confocal microscope with 488/543 nm laser and 63x oil immersion objective. Fluorescein was recorded at excitation 488 nm / emission 410-540 nm and tetramethylrhodamine at excitation 543 nm / 570-600 nm. For R/G imaging three sequential acquisition of the recorded region were performed at 488 nm excitation recording Donor and FRET emission at PMT gains of 830/750/600 V at a pinhole of 228 µm and 1.5 AU.

**Confocal imaging of Alexa 555/Atto 647N siRNA**

Imaging of Alexa 555/ Atto 647N, a red shifted alternative to the analyzed Atto 488/ Atto 590 (4, 5), was performed similar to Atto 488/Atto 590 on the Leica STED CW, with following adjustments: The donor and FRET signal was recorded upon 561 nm excitation at 5 % laser power and the emission was recorded between 570-600 nm and 660-690 nm, respectively, at 100% and 10% HyD detector intensity. Direct excitation of Atto 647N dye was performed at 633 nm with 5% laser power and emission between 660-690nm

**Plasmids**

Plasmids for staining ER, and Golgi apparatus were obtained from Addgene (Cambridge, USA) and were a gift from Michael Davidson. For ER labeling we used the mEmerald-ER-5 plasmid containing the KDEL and Calreticulin sequence (Addgene plasmid # 54083). For Golgi labeling we used the mEmerald-Golgi-7 plasmid containing the GAT sequence (Addgene Plasmid # 54108). Plasmids were transformed and expressed in DH5α *E.coli* cells and purified with the GeneElute Endotoxin-Free Plasmid Maxiprep Kit (Sigma-Aldrich, St. Louis, USA).

**Organelle staining and labeling**

For staining of lysosomes by LysoTracker® Green and Red (both Invitrogen by Thermo Scientic, Waltham, USA), cells were incubated for at least 30 min in culture medium containing 100 nM LysoTracker® dye. Nuclear staining was achieved by 30 min incubation with Hoechst 33342 dye (Sigma-Aldrich) at 1.7 µg/mL. Mitochondrial staining was performed by incubation with 100nM MitoTracker® Green for 30 minutes.

For siRNA transfection following the staining procedure, medium was replaced to dye-free transfection medium as described for siRNA transfection.

In case of lysosome and mitochondria staining after the transfection of siRNA no replacement with dye-free medium was performed.

Golgi and ER plasmids were transfected 1 day prior to analysis with Lipofectamine2000®. For plasmid transfection 10 000 RBE4 cells were seeded one day prior to tranfection in a well of a µ-slide (Ibidi, Munich, Germany) in 200 µL antibiotic free medium. For transfection a mixture of 200 ng plasmid diluted in 50 µL of Optimem (Invitrogen) was mixed with 0.5 µL of Lipofectamine2000® diluted in 50 µL of Optimem and incubation for 20 min before the addition to the cells. Cells were incubated for at least 12 hours to guarantee sufficient expression of the labeled proteins.

### Confocal Imaging of LysoTracker® and nuclear costaining on Zeiss LSM5

siRNA transfected cells, stained before transfection for nuclei and lysosomes, were analyzed on a Zeiss LSM510-UV confocal microscope (Zeiss, Oberkochen, Germany) equipped with 405/488/543 nm lasers and a 63x oil immersion objective. For temperature control, cells cultured on 8-well µ-slides were placed in a multi-well heating system (Ibidi) to guarantee constant 37°C during imaging.

Images were recorded at 512×512 8-bit-pixel resolution with a pinhole of 65 µm and confocal plane depth of 1 µm at z-stacks of 6 slices, resulting in a total image size width and height of 34.95 ×134.95 µm and a depth of 6 µm. The corresponding voxel sizes were 0.26 × 0.26 × 1 µm. The following settings were used to analyze the different stains and dyes: Hoechst 33342: excitation 405/emission 430-480 nm; LysoTracker® Green / Atto488: excitation 488 nm / emission 510-540nm; LysoTracker® Red: excitation 543 nm /emission 580-610 nm; Atto590: excitation 543 nm / emission 605-635nm

### Confocal imaging of organelles on Leica STED CW

MitoTracker® Green, LysoTracker ® Green, Emerald-ER-5 and Emerald-Golgi-7 were analyzed on the Leica CW STED as additional channel in combination with the Alexa 555/ Atto 647N analysis. All organelle dyes and fluorescent proteins were excited at 488 nm at 10% software and 25% hardware laser power. Emission was recorded between 510-540 nm with a normal PMT at a gain of 700 to 900 Volts. Organelles (green), the donor Alexa 555 (red) and FRET signal of Atto 647N were recorded in sequential acquisition mode under live cell conditions.

### R/G ratio processing of FL/TMR

FL/TMR data was R/G analyzed by using the R/G minimal and maximal thresholds of 1 and 2, respectively as earlier described (2, 3)

## SUPPLEMENTARY REFERENCES


1. Roux, F., Durieu-Trautmann, O., Chaverot, N., Claire, M., Mailly, P., Bourre, J.M., Strosberg, A.D. and Couraud, P.O. (1994) Regulation of gamma-glutamyl transpeptidase and alkaline phosphatase activities in immortalized rat brain microvessel endothelial cells. *J Cell Physiol*, **159**, 101-113.
2. Jarve, A., Muller, J., Kim, I.H., Rohr, K., MacLean, C., Fricker, G., Massing, U., Eberle, F., Dalpke, A., Fischer, R. *et al.* (2007) Surveillance of siRNA integrity by FRET imaging. *Nucleic Acids Res*, **35**, e124.
3. Kim, I.H., Jarve, A., Hirsch, M., Fischer, R., Trendelenburg, M.F., Massing, U., Rohr, K. and Helm, M. (2010) FRET imaging of cells transfected with siRNA/liposome complexes. *Methods Mol Biol*, **606**, 439-455.
4. Hirsch, M., Strand, D. and Helm, M. (2012) Dye selection for live cell imaging of intact siRNA. *Biological chemistry*, **393**, 23-35.
5. Krieg, B., Hirsch, M., Scholz, E., Nuhn, L., Tabujew, I., Bauer, H., Decker, S., Khobta, A., Schmidt, M., Tremel, W. *et al.* (2014) New Techniques to Assess In Vitro Release of siRNA from Nanoscale Polyplexes. *Pharm Res*. (doi 10.1007/s11095-014-1589-7; epub ahead of print)

**SUPPLEMENTARY TABLE**

**Table S1**: Parameters for exponential decay model used for integrity calculations

| $\text{degradation level} = A_1 * e^{-\frac{x}{t1}} + A_2 * e^{-\frac{x}{t1}} + y_0$ | |
|---|---|
| $y_0$ | 2.1 ± 0.77 |
| $A_1$ | 61.47 ± 5.75 |
| $t_1$ | 0.08 ± 0.01 |
| $A_2$ | 147.73 ± 18.42 |
| $t_2$ | 0 01 ± 0.00 |
| $R^2$ | 0.998 |

## SUPPLEMENTARY FIGURES

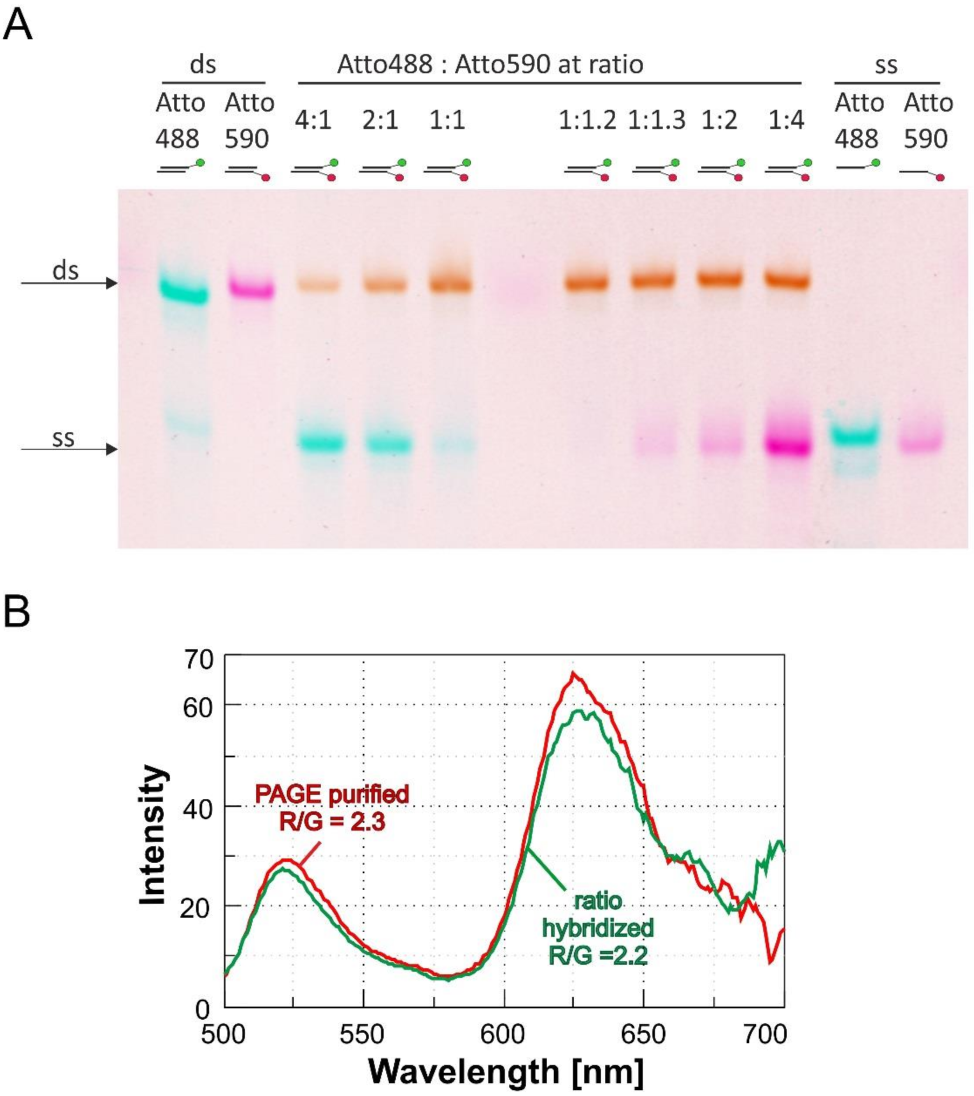


**Figure S1.** siRNA hybridization and spectral characterization. (A) The optimal ratio of sense and antisense strand of the Atto 488/Atto 590 siRNA was analyzed on non-denaturing PAGE by preparing different ratios of donor and acceptor labeled oligonucleotides (as indicated). Complete hybridization is achieved at a ratio of 1:1.2. Arrows indicate single stranded (ss) and double stranded (ds) species. As controls and size marker single labeled duplexes and single strands were chosen. The gel is represented as an inverted overlay of donor, FRET and acceptor signal, where purple color indicates Atto590, blue color indicates Atto488 and red color indicates FRET emission of the dye pair. Figure adapted and based on data published in [4] (B) Spectral profile of hybridized siRNA at the corresponding ratio (green) and siRNA PAGE purified after hybridization (red). Numbers indicate calculated R/G values.

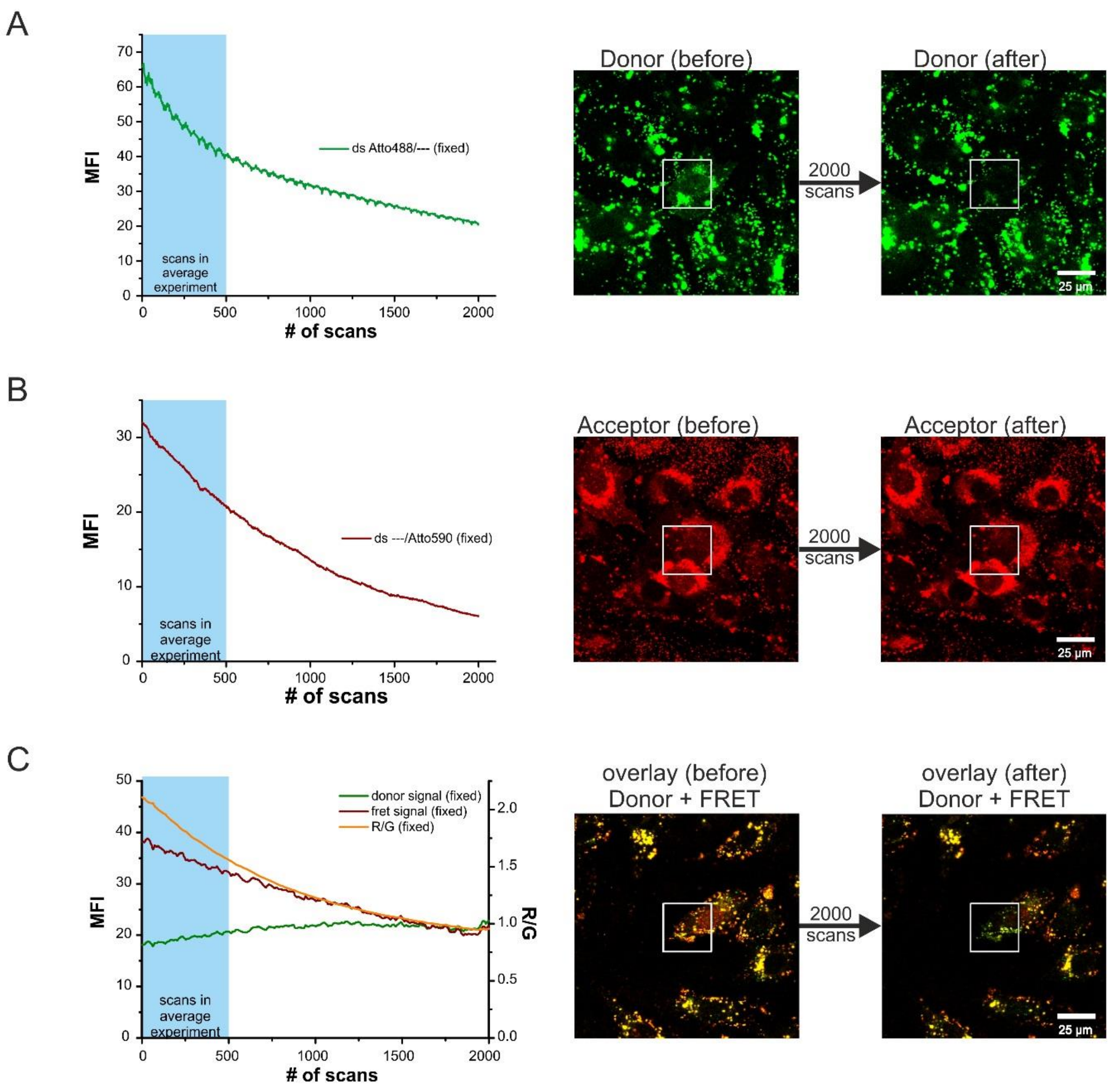


**Figure S2**. Susceptibility of labeled siRNA to bleaching during confocal observation. RBE4 cells transfected with donor only (A), acceptor only (B) and FRET labeled siRNA (C) were fixed and imaged for up to 2000 scans with a CLSM, corresponding to 33 h of constant imaging in the R/G imaging mode. White rectangles in images on the right indicate the region that were permanent excited and analyzed for determination of donor (green), acceptor (red) or FRET (red) MFI signal for the respective channel. R/G ratio of FRET siRNA is depicted in orange (C). Scale bar 25 µm.

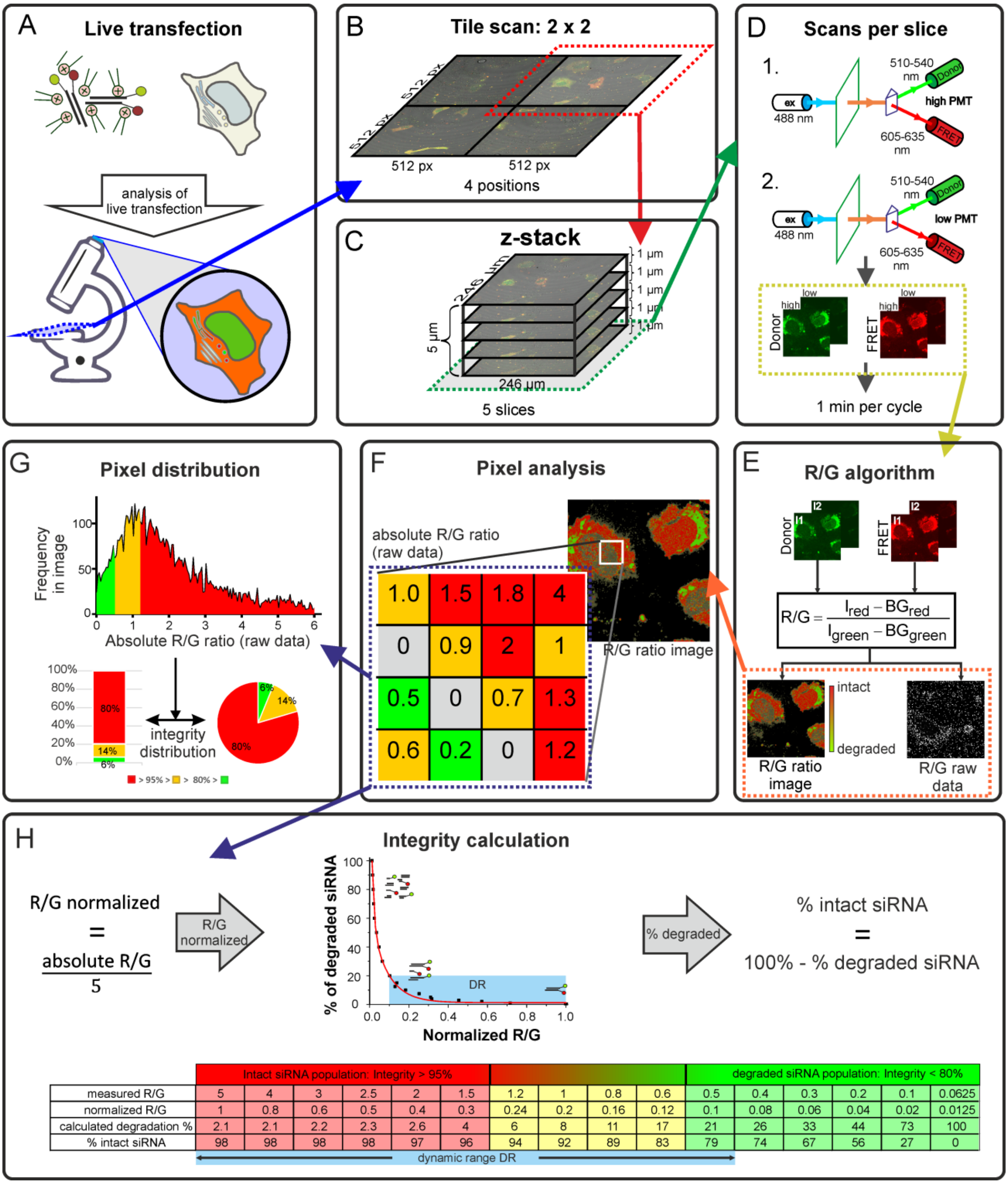


$$R/G = \frac{I_{red} - BG_{red}}{I_{green} - BG_{green}}$$

| | Intact siRNA population: Integrity > 95% | | | | | | | | | | degraded siRNA population: Integrity < 80% | | | | | |
|---|---|---|---|---|---|---|---|---|---|---|---|---|---|---|---|---|
| measured R/G | 5 | 4 | 3 | 2.5 | 2 | 1.5 | 1.2 | 1 | 0.8 | 0.6 | 0.5 | 0.4 | 0.3 | 0.2 | 0.1 | 0.0625 |
| normalized R/G | 1 | 0.8 | 0.6 | 0.5 | 0.4 | 0.3 | 0.24 | 0.2 | 0.16 | 0.12 | 0.1 | 0.08 | 0.06 | 0.04 | 0.02 | 0.0125 |
| calculated degradation % | 2.1 | 2.1 | 2.2 | 2.3 | 2.6 | 4 | 6 | 8 | 11 | 17 | 21 | 26 | 33 | 44 | 73 | 100 |
| % intact siRNA | 98 | 98 | 98 | 98 | 97 | 96 | 94 | 92 | 89 | 83 | 79 | 74 | 67 | 56 | 27 | 0 |

dynamic range DR

**Figure S3.** Schematic representation of experimental workflow for live cell imaging, R/G ratio analysis and integrity calculation. (A) Live cell observation of RBE4 cells on a CLSM and real-time transfection. (B) Setup for a 2x2 tile scan with 4 scanning positions each at 512x512 pixels (see Movie M1 as example). (C) z-stack acquisition over a distance of 4 µm with 5 slices at 1 µm section thickness and XxY dimension of 246x246 µm. (D) Microscopy setting for R/G ratio imaging mode with two similar scans at different PMT settings (i.e. high and low) resulting in total in 1 min acquisition intervals between two scans. (E) R/G ratio algorithm of Donor and FRET images at the different acquired PMT settings (i.e. I1 and I2). After Thresholding and background (BG) subtraction the R/G ratio is depicted in the R/G ratio image with red pixels indicating intact siRNA (R/G ≥ 1.2) and green pixels indicating degraded siRNA (R/G ≤ 0.5). R/G values between 1.2 and 0.5 are color coded by a transition from green to red (olive). (F) For the pixel analysis the R/G ratio of each pixels is extracted from the R/G raw data. Example depicts the color coding according to R/G thresholds of 1.2 and 0.5. (G) A histogram of the pixel distribution can be generated and pixels are classified as degraded (green), intermediate (olive) and intact (red). (H) Integrity calculation on basis of R/G value from R/G ratio algorithm. The image R/G (absolute value) is normalized by dividing by

5 (average R/G of intact siRNA in cuvette and image data) and degradation level is calculated according to R/G calibration model and used to determine the integrity level. The table in the lower part of the box depicts typical R/G values and integrity levels. The blue bar indicates dynamic range (DR) of the R/G system.

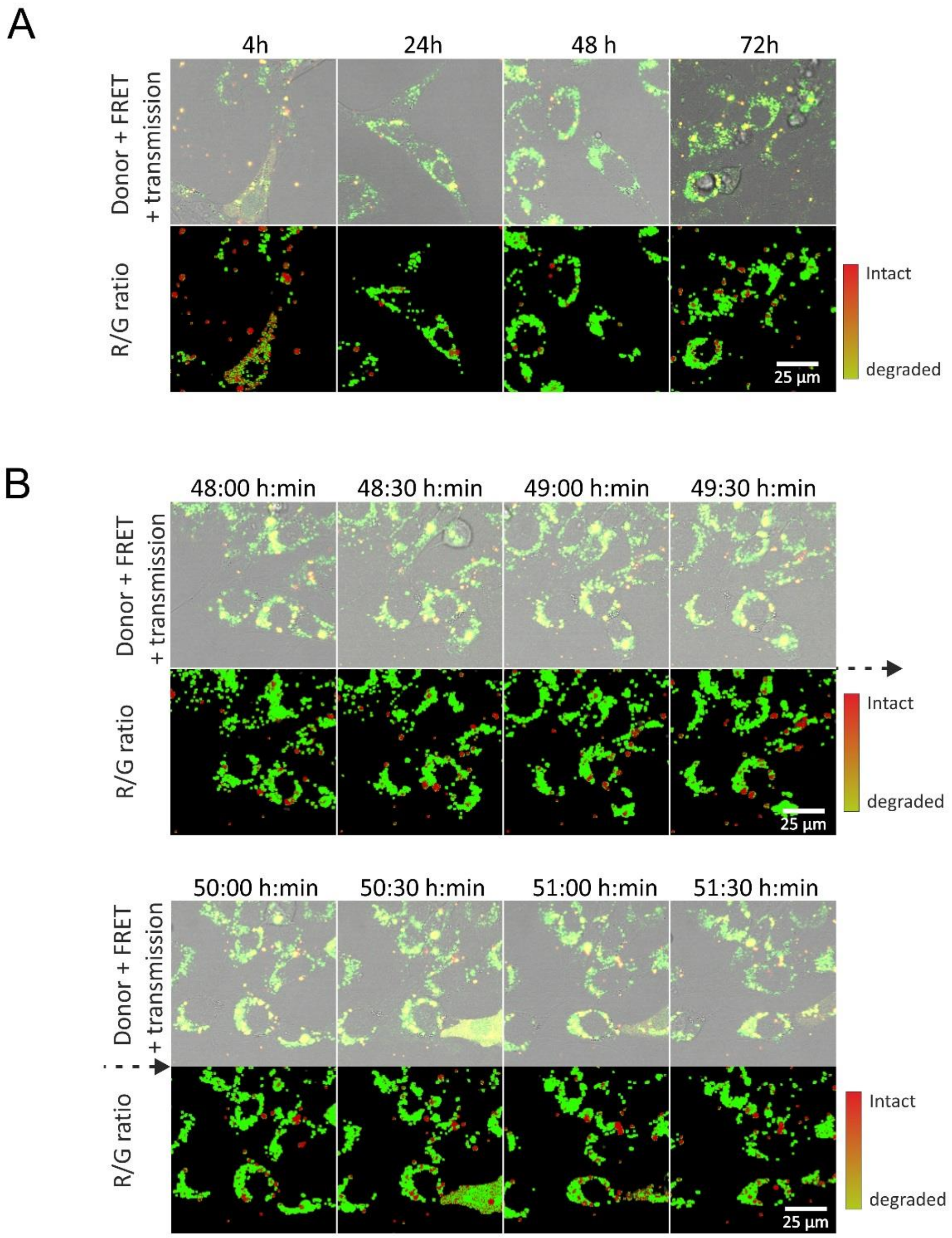


**Figure S4.** Long-time observation of transfected Atto 488/Atto 590 siRNA. (A) RBE4 cells 4 h, 24 h, 48 h and 72 h after transfection. Depicted are RGB overlay (upper row) of source data (donor, FRET, transmission) and the R/G ratio image (lower row). Scale bar 25 µm. (B) Image sequence of cells after 2 days of being transfected with Atto 488/Atto 590 siRNA. Cells were observed for up to 5 hours and sequence depicts snapshots every 30 min (see Movie M6). Images show overlay of donor/FRET/transmission signal (upper row) and R/G ratio image (lower row). Scale bar 25 µm.

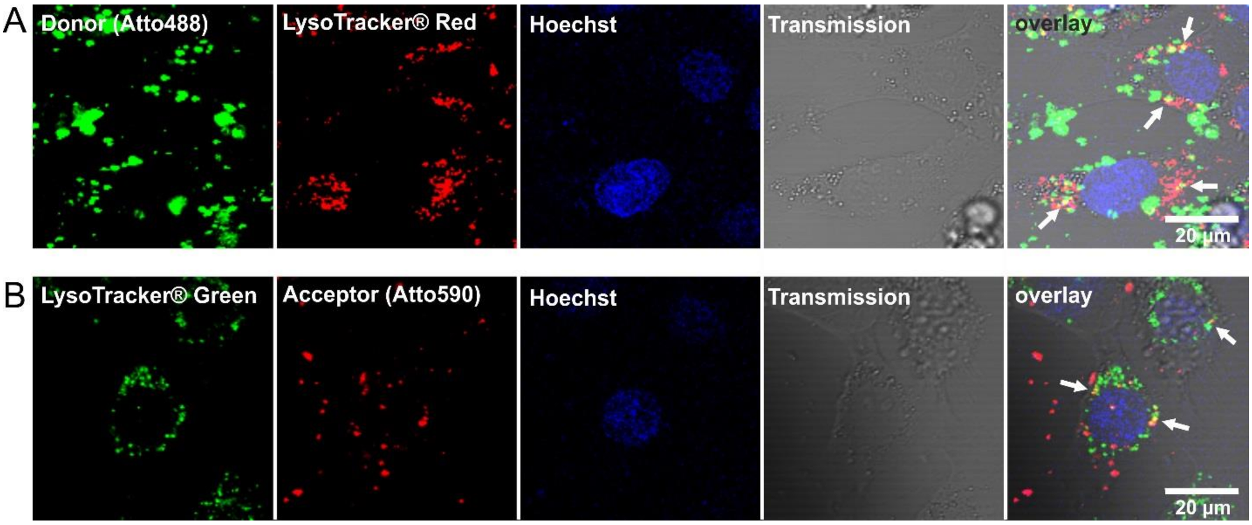


**Figure S5.** Colocalization of lipoplexes with stained lysosomes. RBE4 cells transfected with single labeled siRNA by simultaneously staining of the nucleus with Hoechst day and the lysosomes with LysoTracker®. (A) Atto488 labeled siRNA with costaining of lysosomes by LysoTracker® RED. Colocalization can be observed by yellow spots (indicated by white arrows). (B) Atto 590 labeled siRNA with costaining of lysosomes by LysoTracker® Green (colocalization indicated by white arrow). Scale bar 20µm.

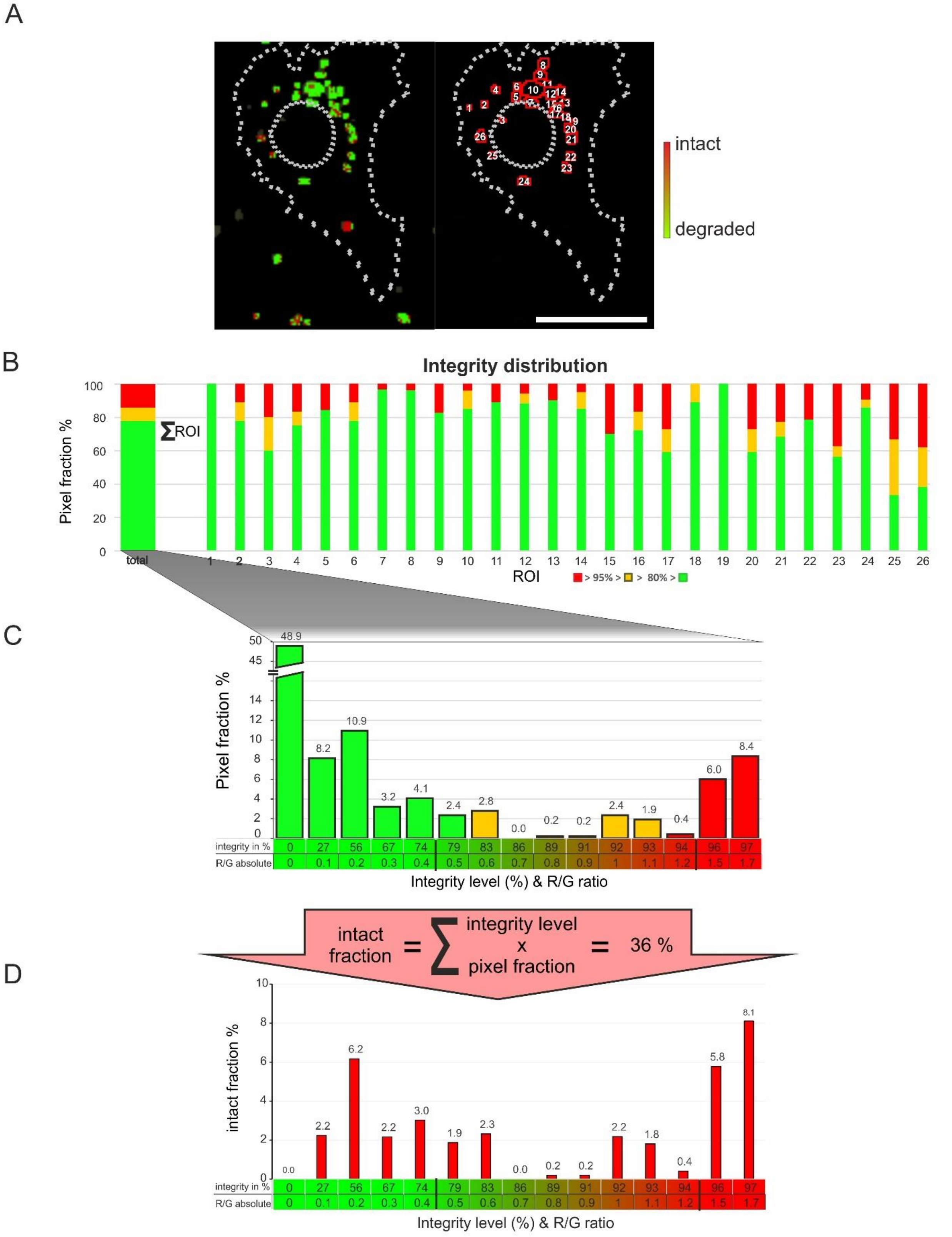


**Figure S6.** Analysis of perinuclear aggregations after depletion of released siRNA. (A) Cell after depletion as R/G ratio image (left) and ROI-mask (black and white image; right). ROIs for single aggregates are highlighted in red displaying the number of the respective ROI. Scale bar 25µm. (B) Analysis of the single ROIs (1-26) and the sum of all ROIs (total) as integrity distribution on pixel basis. Thresholds for intact, intermediate and degraded state according to R/G algorithm threshold, i.e. 1.2 and 0.5. (C) Pixel fraction of the total ROI assigned to their R/G value and integrity level, respectively. (D) The fraction of intact siRNA is calculated as a sum of pixel fraction per R/G value times calculated integrity level. Intact fractions for each R/G value and integrity level are depicted in the lower graph, resulting in a total of 36% of intact siRNA.

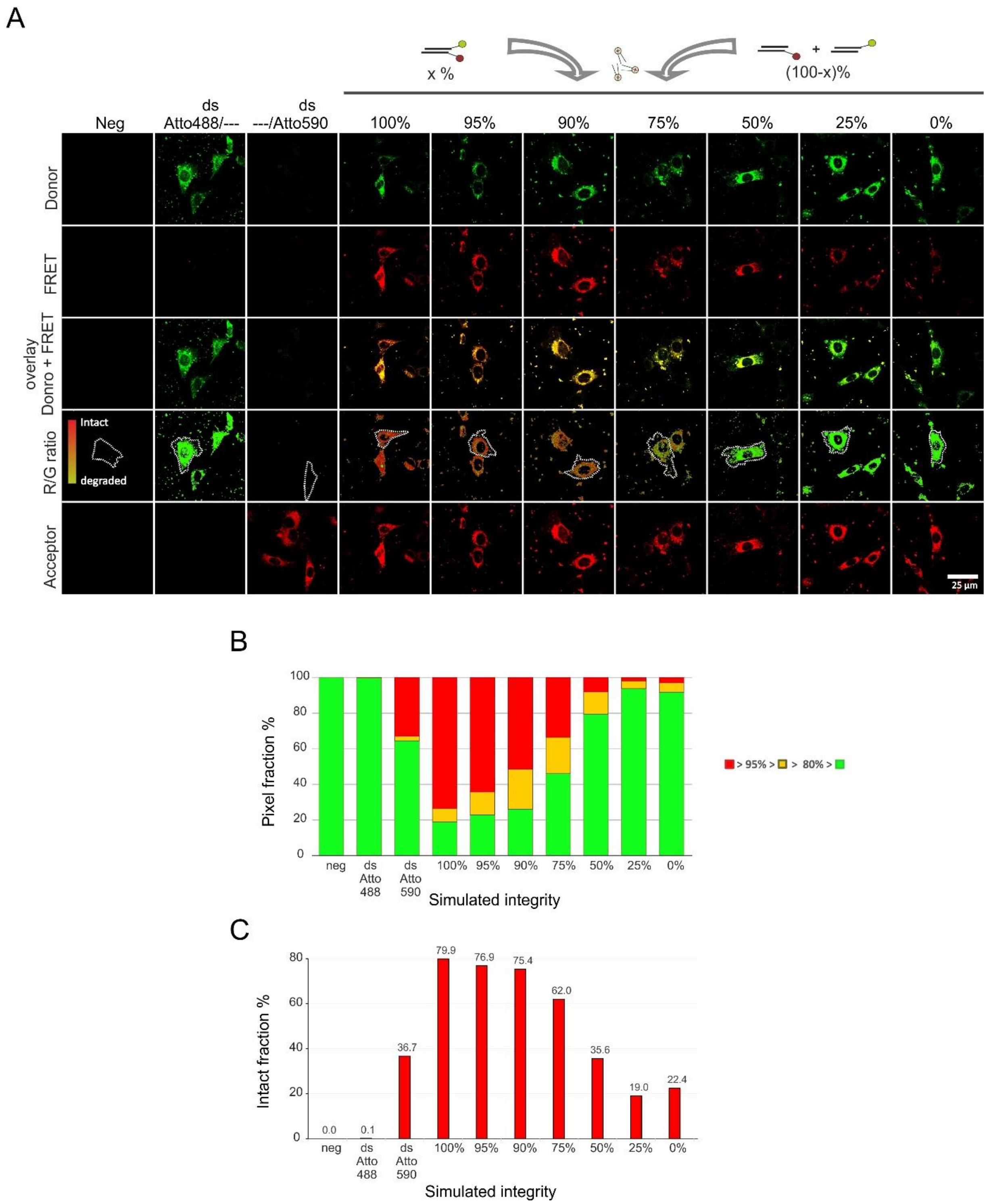


**Figure S7.** Integrity simulation and R/G processing inside fixed cells with Attot488/Atto590 siRNA. (A) Depicted are the donor, FRET, donor/FRET-overlay, R/G ratio and acceptor signal of fixed RBE4 cells transfected with samples referring to different siRNA integrity states of Atto488/Atto590 siRNA. The samples referring to 100 %, 95 %, 90 %, 75 %, 50 %, 25 % and 0 % intact siRNA are obtained by mixing different amounts of single labeled duplexes (simulating degraded siRNA) and double labeled (referring to intact siRNA) duplexes. Dotted lines in R/G ratio image highlight cells for further analysis. Scale bar indicates 25 µm. (B) Integrity distribution on pixel basis in displayed region. (C) Fraction of intact siRNA in simulated samples and regions, respectively.

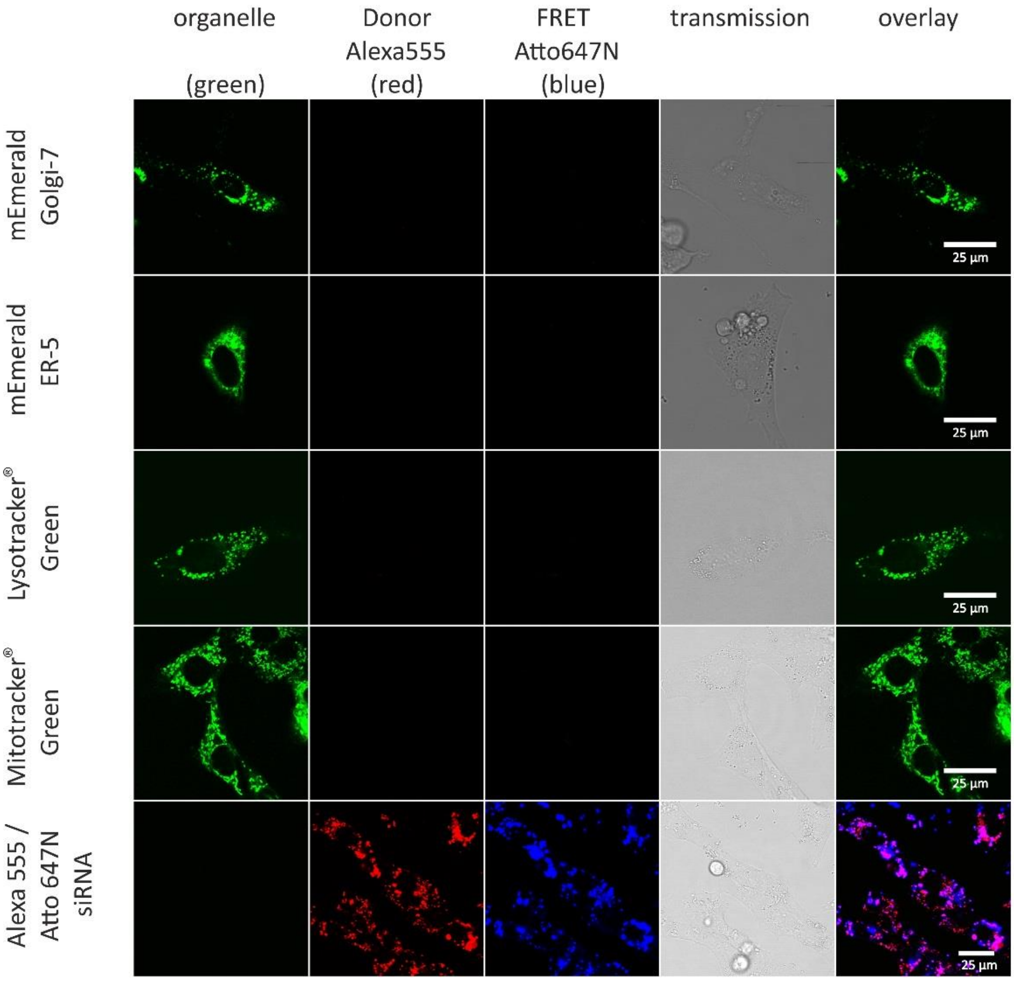


**Figure S8.** Alexa 555 / Atto 647N siRNA and different organelle markers for colocalization studies. Recorded were the organelle signal (green), donor signal of Alexa 555 (red) and the FRET signal of Atto 647N (blue). As organelle markers MitoTracker® Green, LysoTracker® Green and mEmerald tagged proteins specific for ER (mEmerald-ER-5) and Golgi apparatus (mEmerald-Golgi-7). Data was recorded in sequential mode allowing to discriminate between the different signals. Scale bar 25 µm.

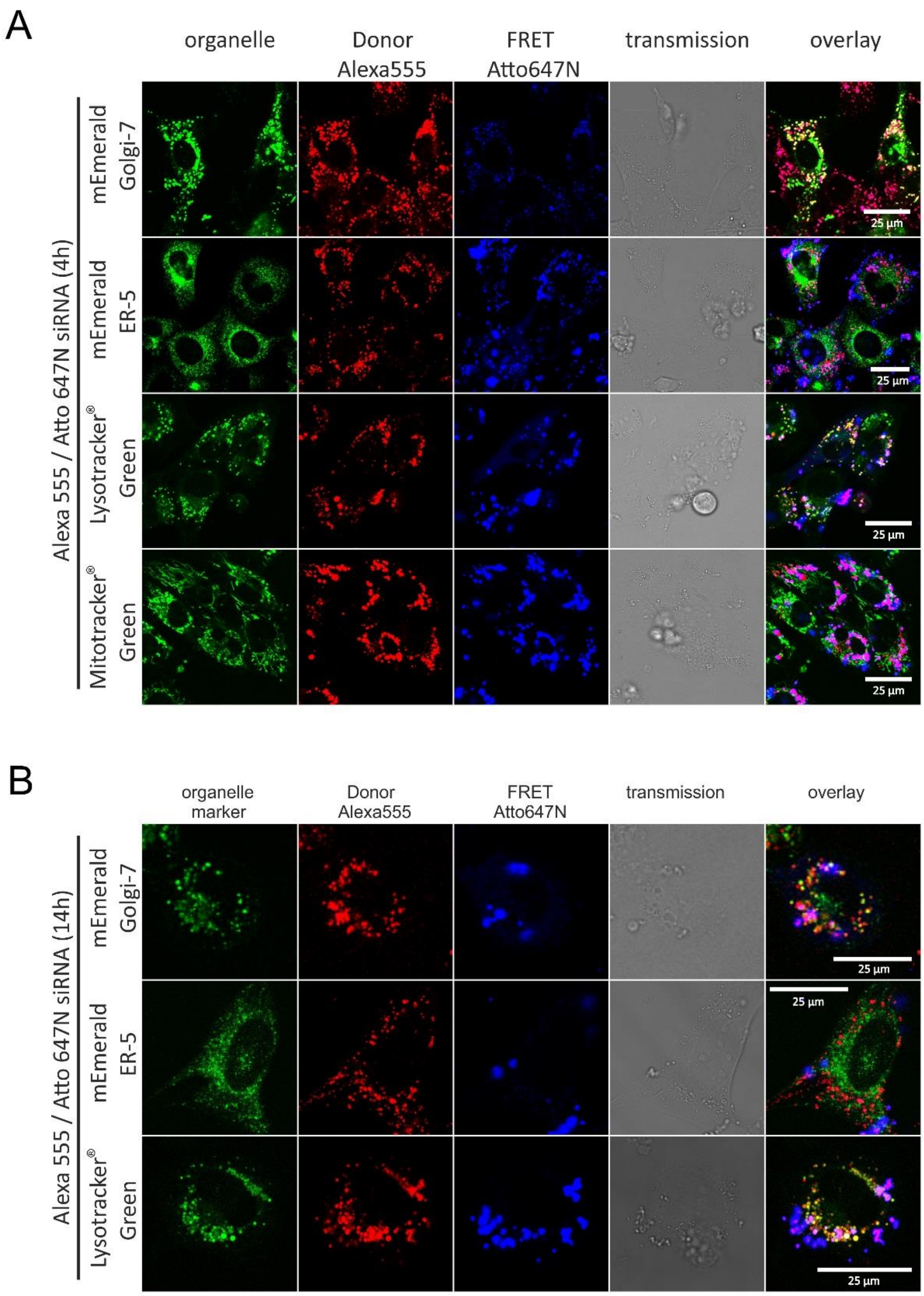


**Figure S9.** Alexa 555 / Atto 647N siRNA and different organelle markers in colocalization studies. Sequences depict expressed mEmerald-Golgi-7 (1st row) and mEmerald-ER-5 (2nd row) proteins after 4 h (A) and 14 h (B) incubation with Alexa 555 / Atto 647N siRNA lipoplexes. In addition LysoTracker® Green (3rd row) and MitoTracker® Green (only 4h) were analyzed. Colocalization is represented by yellow and white pixels in the overlay image. Scale bar 25 µm.

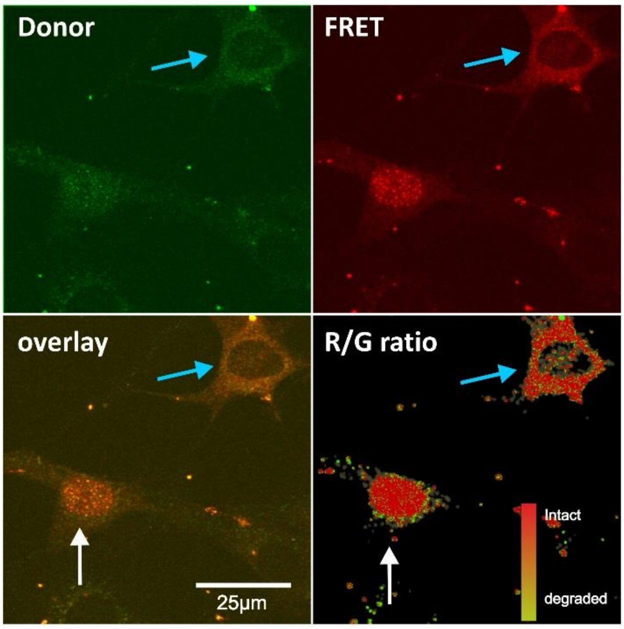


**Figure S10.** Nuclear accumulation of FL/TMR labeled siRNA in fixed cells. RBE4 cells after transfection showing siRNA accumulation mainly in the cytoplasm (light blue arrow) or in the nucleus (white arrows). Nuclear siRNA shows additionally accumulation in small distinct foci. Sequence depicts donor signal (upper left), FRET signal (upper right), overlay of donor and FRET signal (lower left) and R/G ratio processing (lower right). Scale bar 25 µm.

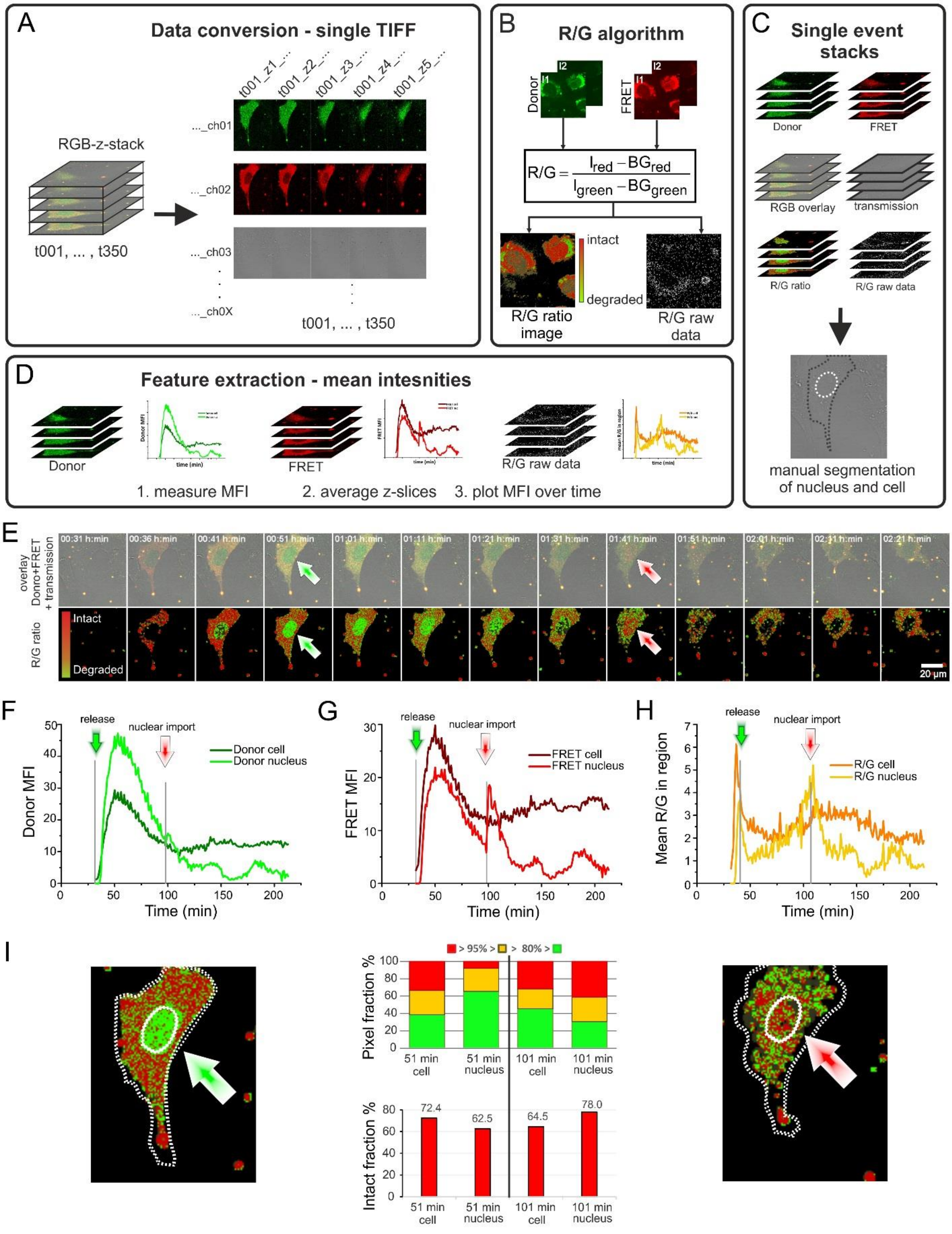


**Figure S11.** Schematic representation of the workflow for single cell analysis with example of a class F event. (A) Live cell observation data of Atto488/Atto590 transfection is separated into single in single TIFF-files with coding for recorded channels, z-position, tile-position and timeframe. (B) R/G ratio processing by calculating the R/G ratio on the basis of donor and FRET channel, resulting in R/G ratio image and an R/G raw data file. (C) For each signal, i.e. donor, FRET, transmission, R/G ratio image a xyzt-stack is build and used for merging the donor-FRET-transmission RGB overlay. Raw data is imported and also built to an xyzt-stack. For each release event a subregion of the original stack is cropped and used for further analysis (event-ROI). With help of the substacks segmentation of the nucleus and cell outline is manually performed. (D) For nucleus and complete cell ROI, the MFI values are measured for each timeframe of a z-slice. The final graph depicts z-averaged MFI values or the mean R/G value for a single event and fluorescence

channel. (E) Image sequence of class F event as seen in Figure 3F & Movie M4 as RGB overlay of source data and R/G ratio image. Green shaded arrow indicates green dominated nuclear signal and red shaded arrow nuclear import of intact siRNA. Scale bar 25 µm. (F-H) Donor MFI (F), FRET MFI (G) and R/G mean value (H) in cell and nuclear region. Green arrow indicates time point of release, red shaded arrow time point of nuclear import of intact siRNA. (I) Pixel based integrity distribution (upper graph) and fraction of intact siRNA (lower graph) of cell displaying mainly green (degraded) or red (intact) R/G pixel inside the nucleus. Cell and nuclear region are highlighted by dotted lines.

**SUPPLEMENTARY MOVIES**

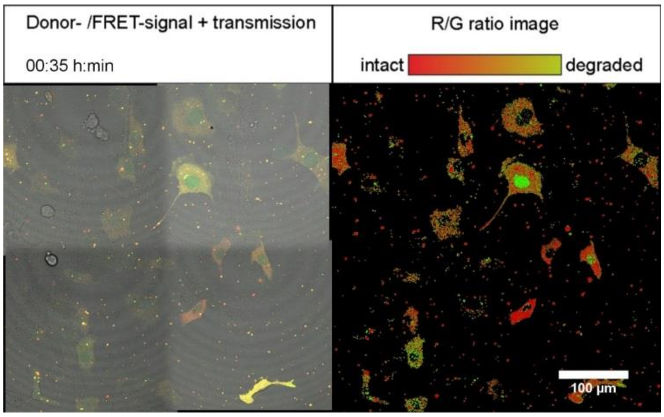


**Movie M1.** Movie of one live cell experiment acquired as 2x2 tile scan and stitched to one data set. Multiple cells displaying all types of event classes can be seen at 2 min intervals. Images show overlay of donor/FRET/transmission signal (left) and R/G ratio image (right). Starting direct after addition of transfection mixture to cells, the movie is displayed at 5 fps for an observation period of 350 min. Scale bar 100 µm.

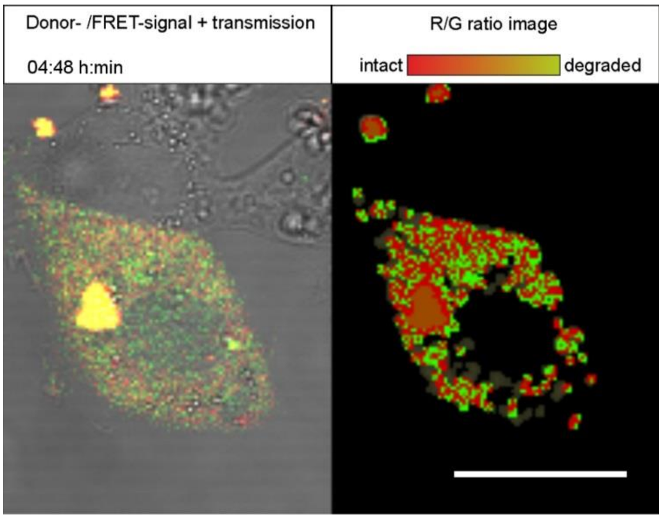


**Movie M2.** Movie of cell displaying a class A (uptake of lipoplex) and B event (release) at 50 s intervals. Images show overlay of donor/FRET/transmission signal (left) and R/G ratio image (right). Starting at 3:50 h:min after transfection, movie is displayed at 5 fps for an observation period of 74 min. Scale bar 25 µm.

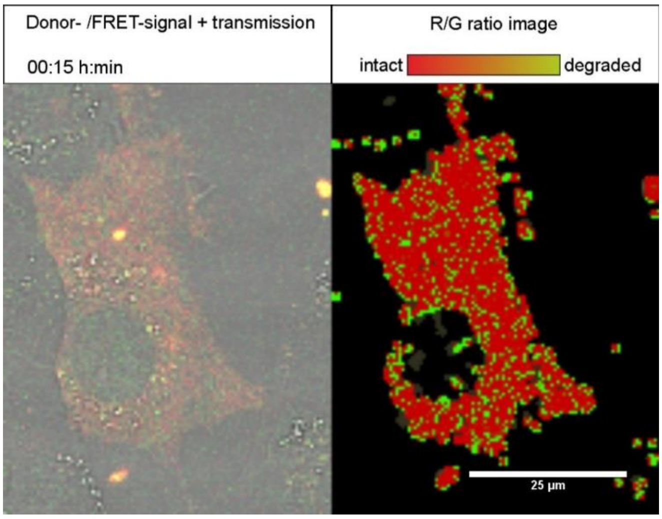


**Movie M3.** Movie of cell displaying class B (release), C (depletion) and D (perinuclear accumulation) event at 2 min intervals. Images show overlay of donor/FRET/transmission signal (left) and R/G ratio image (right). Starting direct after addition of transfection mixture to cells, the movie is displayed at 5 fps for an observation period of 350 min. Scale bar 25 µm.

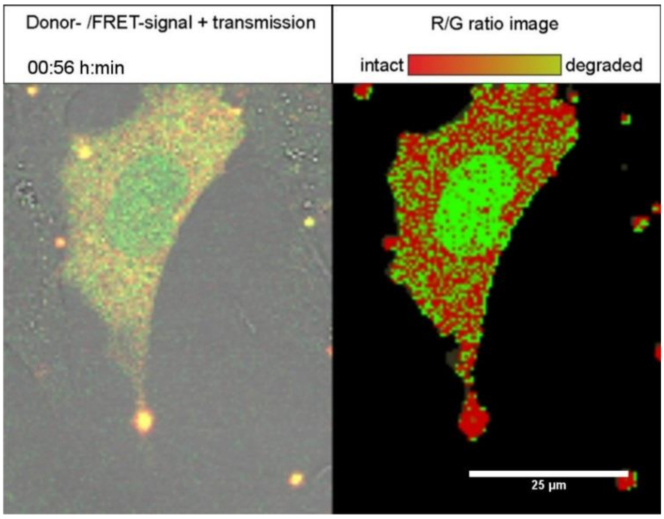


**Movie M4.** Movie of cell displaying class B (release), C (depletion), F (nuclear import of intact siRNA), D (perinuclear accumulation) and (E) (secondary release) event at 2 min intervals. Images show overlay of donor/FRET/transmission signal (left) and R/G ratio image (right). Starting direct after addition of transfection mixture to cells, the movie is displayed at 5 fps for an observation period of 350 min. Scale bar 25 µm.

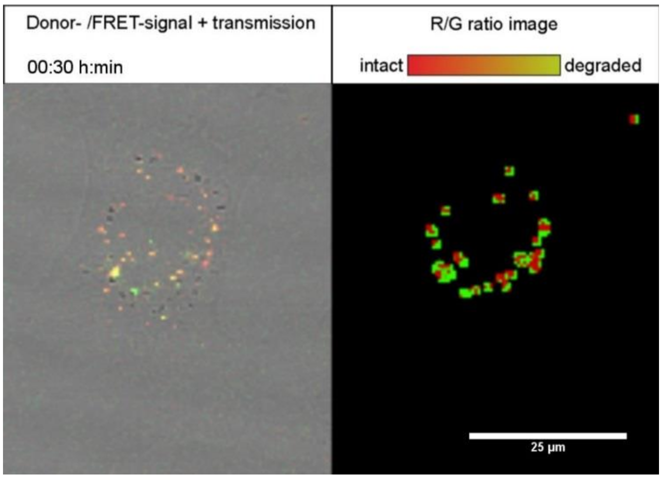


**Movie M5.** Movie of cell displaying class G event (progression of siRNA degradation inside endocytotic structures) at 2 min intervals. Images show overlay of donor/FRET/transmission signal (left) and R/G ratio image (right). Starting direct after addition of transfection mixture to cells, the movie is displayed at 5 fps for an observation period of 150 min. Scale bar 25 µm.

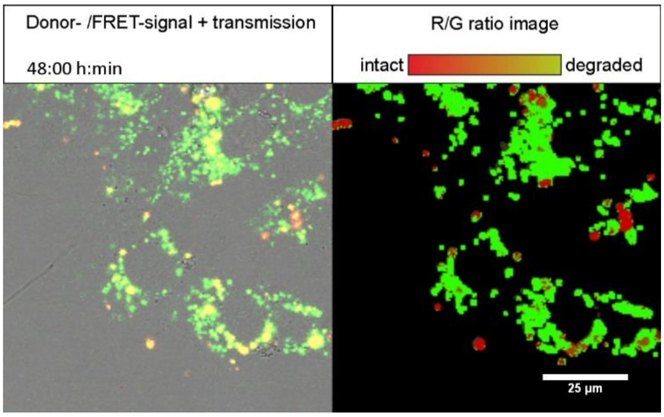


**Movie M6.** Movie of cells after 2 days of being transfected with Atto 488/Atto 590 siRNA at 2 min intervals. Images show overlay of donor/FRET/transmission signal (left) and R/G ratio image (right). Starting 48 h after the addition of transfection mixture to cells, the movie is displayed at 5 fps for an observation period of 4 h. Scale bar 25 µm.
